# Carbon Dot-Based Electrochemical and Optical Sensors for Pharmaceutical Analysis and Point-of-Care Diagnostics

**DOI:** 10.3390/bios16050246

**Published:** 2026-04-28

**Authors:** Ganesh Gollavelli, Chiranjib Patra, Chiranjeevi Korupalli, Manuri Brahmayya, Yong-Chen Ling

**Affiliations:** 1 Department of Chemistry, Aurora Deemed to be University, Hyderabad 500098, India; 2 Department of Chemistry, National Tsing Hua University, Hsinchu 300044, Taiwan; 3 Department of Biological Sciences, School of Engineering and Sciences, SRM University-AP, Amaravati 522502, India; 4 Department of Chemistry, Vallurupalli Nageswara Rao Vignana Jyothi Institute of Engineering and Technology, Hyderabad 500118, India

**Keywords:** nanobiosensors, carbon dots, carbon quantum dots, pharmaceutical analysis, electrochemical sensing, optical sensing, point-of-care testing

## Abstract

Because of their special optical and electrochemical characteristics, superior biocompatibility, adjustable surface chemistry, and inexpensive, scalable synthesis, carbon dots (CDs), including carbon quantum dots and graphene quantum dots, have become powerful and adaptable nanomaterials for advanced pharmaceutical analysis and other toxicants. The sensitive and selective detection of active pharmaceutical substances, degradation products, contaminants, biomarkers, and therapeutic medication levels in complex matrices has shown great promise in recent years with CD-based nanobiosensors. The development of various sensing platforms, such as electrochemical, optical, and dual-mode biosensors, as well as integration into microfluidic, paper-based, and wearable point-of-care (POC) devices, is made possible by their intrinsic fluorescence, effective electron transfer capacity, and ease of functionalization. With an emphasis on sensing mechanisms, biorecognition techniques, and analytical performance, this study critically reviews current developments in CD-based nanobio/chemosensors for pharmaceutical analysis. It includes a thorough discussion of important applications in drug development, stability research, therapeutic drug monitoring, and drug quality control. Along with new developments like green synthesis, AI-assisted signal processing, and smart sensing platforms, current issues with reproducibility, standardization, biocompatibility, and regulatory validation are highlighted. Lastly, prospects for the industrial application and clinical translation of CD-based nanobiosensors are discussed.

## 1. Introduction

Human health and ecosystems are under serious threat due to the rapid industrialization and adoption of synthetic chemicals and their continuous release into the environment at trace levels [1]. Detecting their level of release and accurate identification of the chemical compounds onsite have been daunting [2]. Although chemo-biosensor-based technology has reached a certain level of advancement, it still faces critical issues that require immediate attention: (a) ultra-low levels of detection of chemicals in a complex environmental matrix; (b) though they perform well on a lab scale, in real conditions, they show lower sensor stability and reproducibility; and (c) on-site rapid analysis without sophisticated instrumentation [3,4]. The rapid, simultaneous detection of trace amounts of contaminants in air, water, food, and soil became a very big challenge to the traditional chemo- or biosensor systems [5,6].

In order to address these challenging issues, chemo and biosensors have emerged as a novel instrument with exceptionally great features by the integration of nanotechnology and biological sensing for the detection and analysis of various biological and chemical entities, exhibiting high sensitivity and specificity [7,8,9]. Because of their small size (10^−9^ m), nanobiosensors contain an unparalleled advantage over their conventional analogs. The increased surface reactivity, quantum effects, and high surface-to-volume ratio enabled analyte surface interactions, and fast response times have been great advantages in modern sensors [10,11]. The potential of nanobiosensors has been adopted in a wide range of applications, including environmental monitoring, medical diagnosis, and health or disease monitoring [12,13].

Among the plethora of nanomaterials, such as metals, metal oxides, and different carbon-based nanomaterials, CDs have displayed a prominent role in analytical science [13,14]. The CDs are simple, facile, and economical to fabricate, with unique optical properties and tunable emissions. Moreover, they are easy to functionalize and show greater biocompatibility [15]. As emerging nanomaterials, the CDs are demonstrated as a potential scaffold for chemo and biosensing applications. The unique features of these allotropes of carbon nanomaterial have been of interest to biosensors, as they have shown good sensitivity, rapid results, and enabled real-time research [16,17].

**Figure 1:**
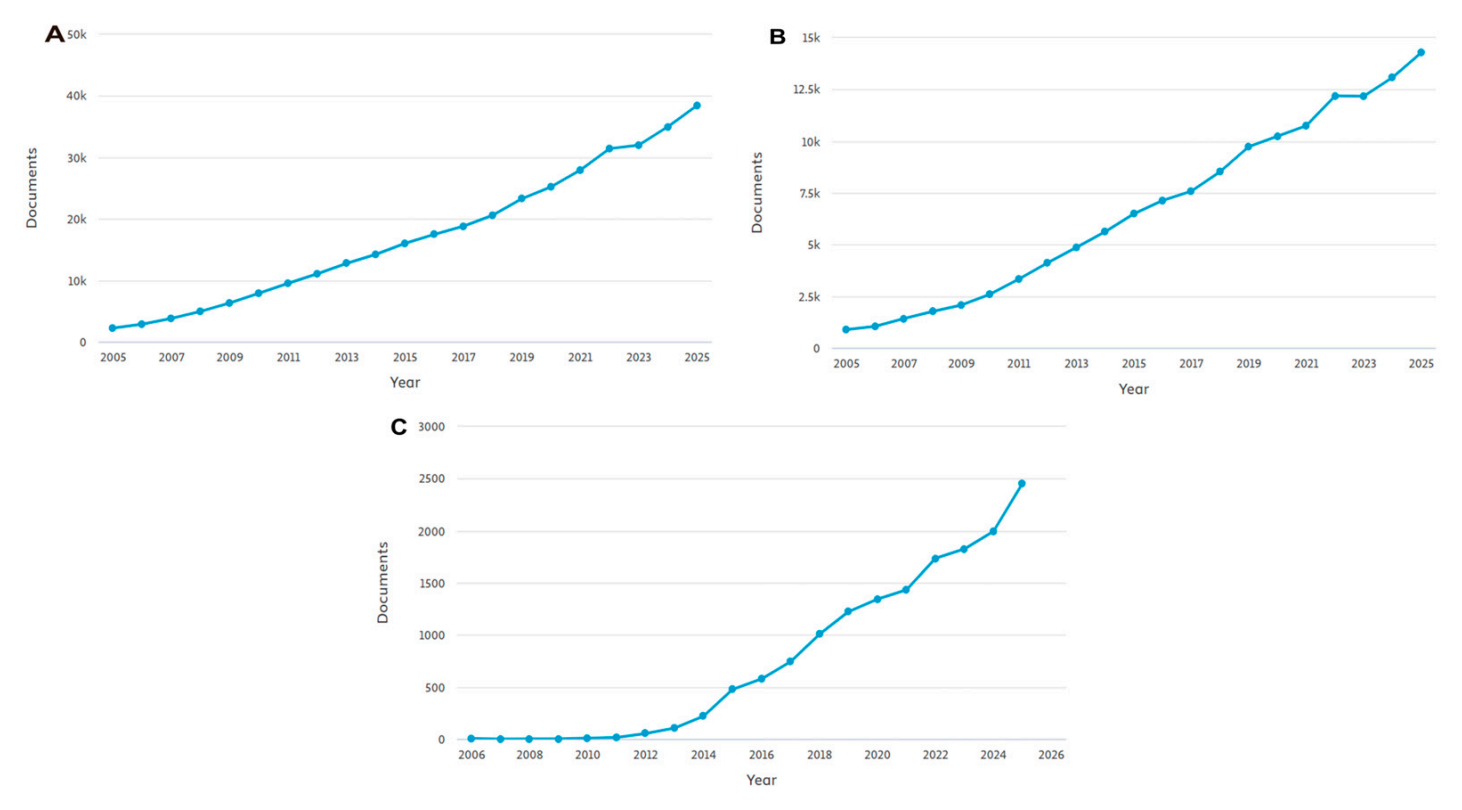
Publication statistics related to the topic. (**A**) Nanomaterials for biosensors. (**B**) Carbon-based nanomaterials for biosensors. (**C**) CDs for biosensors. The statistics have been taken from Scopus from 2005 to 2025.

The current statistics on biosensors have shown a rapid growth trend over the past 20 years. About 362,129 results have been observed in Scopus by giving the keywords nanomaterials and biosensors (see Figure 1A). For carbon nanomaterial and biosensor keywords, we have received 139,982 results (see Figure 1B); for exclusively on CD and biosensor keywords, we have received 15,234 articles (Figure 1C), emphasizing the importance of the field. However, the rsearch remains in the proof-of-concept stage due to the lower stability and reproducibility in real-time complex sample analysis [17,18].

**Scheme 1:**
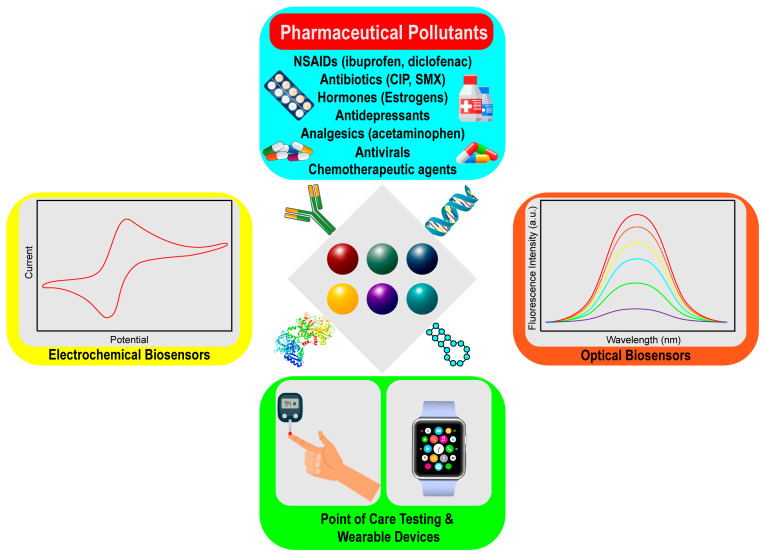
CD-based biosensors based on electrochemical, optical, and point-of-care detection of various pharmaceutical pollutants.

There are different types of nanobiosensors discussed in the literature. Namely, electrochemical sensors, optical sensors, fluorescent sensors, and point-of-care sensors for various applications (see Scheme 1) [1,19,20,21,22,23,24]. However, the topic is as follows: “CD-based hybrid chemo and nanobiosensors to identify various advanced pharmaceutical products and other pollutants by electrical, optical, fluorescent, point-of-care validation, smartphone, and AI-assisted validation, which is still lacking”. Hence, in this review, we discussed, from the synthesis and characterization of CDs to the types of sensors, their mechanism, stability, biorecognition elements, and functionalization strategies, recent developments, challenges, limitations, and future perspectives.

## 2. Biosensors

Chemo and biosensors are emerging devices that are gaining popularity in analytical research globally. In biosensors, biological response material (enzyme, aptamer, DNA or antibody, cell, and tissue) with transducers (electrochemical, optical, thermal, gravimetric, and acoustic) is used to specifically measure biological or chemical analytes by transforming a biological signal into a quantifiable electrical signal.

**Figure 2:**
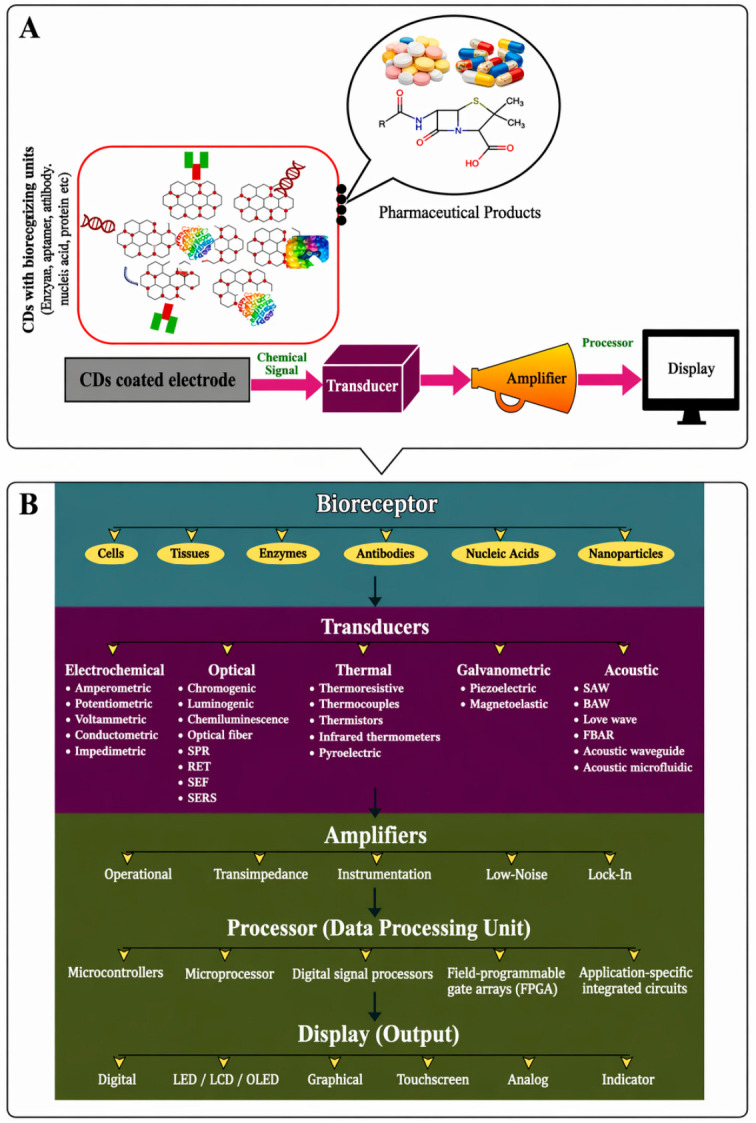
(**A**) Schematic representation of a CD-coated electrode for biosensing applications and the signal processing system. (**B**) Detailed sketch of the components of biosensors.

The four basic parts of a typical biosensor are shown in Figure 2A: (1) CDs or nanomaterial-coated electrodes contain sensor receptors; (2) a signal transducer that perceives changes in one or many different types of signals, including impedance, electrical current, light, and optical density; (3) a signal processing unit; and (4) an amplifier that produces clear outputs for analyzing the phenomenon and interpretation, and a sensible bioelement that identifies the target analyte [25]. Figure 2B shows the detailed information about the general biosensors [26].

Traditionally, the most frequently used electrode materials are gold and carbon. These materials are favored by researchers because of their stability, biocompatibility, and favorable electron transfer kinetics. Due to their lower sensitivity and selectivity, electrochemical detection of trace analytes is frequently unachievable on unaltered surfaces [27]. To get around this problem, nanomaterials have been incorporated into the electrode surfaces. The redesigned electrodes provide great selectivity in the presence of interferences. Nanocarbons, nanoparticles (NPs), conductive polymers, and nanocomposites are among the nanostructured materials that have been identified as being able to refine and sensitize electrode surfaces [28].

## 3. Carbon Dots: Structure, Properties, and Synthesis

### 3.1. Structure and Classification of CDs

Numerous scientific disciplines are showing a great deal of interest in CDs, a kind of 0D carbon-based nanomaterial. CDs often feature nanocrystalline cores with a graphitic structure. It is composed mainly of sp^2^ hybridized carbon. There have also been sporadic reports of diamond-like sp^3^-hybridized carbon cores. Since being identified as a new member of the “carbon family” in the early 2000s, CDs have rapidly gained widespread recognition due to their unique characteristics. The development of CDs occurred in three phases: 1. the discovery period from 2004 to 2006; 2. the initial development phase from 2007 to 2011; and 3. the rapid expansion phase from 2011 to the present [29,30]. In the year 2004, Xu and his researchers discovered the formation of bright light illuminating CDs during the purification process of single-walled carbon nanotubes (SWCNTs) [31]. However, in 2006, Sun and his associates started official usage of the term “carbon quantum dots” and initiated a period of intense CD research and development as well as global interest in this field [30,32].

**Figure 3:**
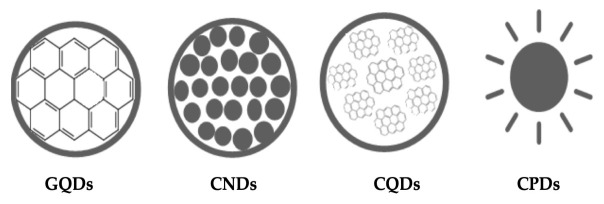
Types of CDs: GQDs, CNDs, CQDs, and CPDs.

CDs are classified according to their properties, core of the carbon structure, and functional groups on the surface. Figure 3 illustrates the various forms of CDs, including carbon quantum dots (CQDs), graphene quantum dots (GQDs), carbon nanodots (CNDs), and carbonized polymer dots (CPDs). The CQDs are crystalline nanospheres with intrinsic state luminescence and quantum confinement effect (QCE). These properties stem from a plethora of chemical functional groups on the CD surface. GQDs are essentially anisotropic graphene fragments that are stacked in one or more layers of graphene sheets. The GQDs show quantum confinement and edge effects due to the presence of various chemical functionalities within their interlayer defects and on their edges. Without revealing their crystalline or polymeric structures, the CNDs possess a high degree of carbonization along with edge effects. Additionally, CNDs do not exhibit the QC effect. Carbon crosslinked nanohybrids and polymer aggregates are the CPDs. The CPDs contain a carbonized core at the center covered by either the polymeric chains or functional groups [33].

### 3.2. Physicochemical Properties Relevant to Biosensing

#### 3.2.1. Photoluminescence and Fluorescence Mechanisms

The physical and chemical properties of CDs must be taken into account when using them for sensing applications in order to meet the requirements of the sensing application. Because of their small size and ability to emit fluorescence as a sensing signal, CDs are highly useful as sensing receptors in nanoprobes. The environment has a direct impact on CD fluorescence. CD fluorescence is either enhanced or quenched when they interact with analytes. CDs must be modified using chemical and physical methods in order to improve the fluorescence performance in accordance with the particular analytes [34].

**Figure 4:**
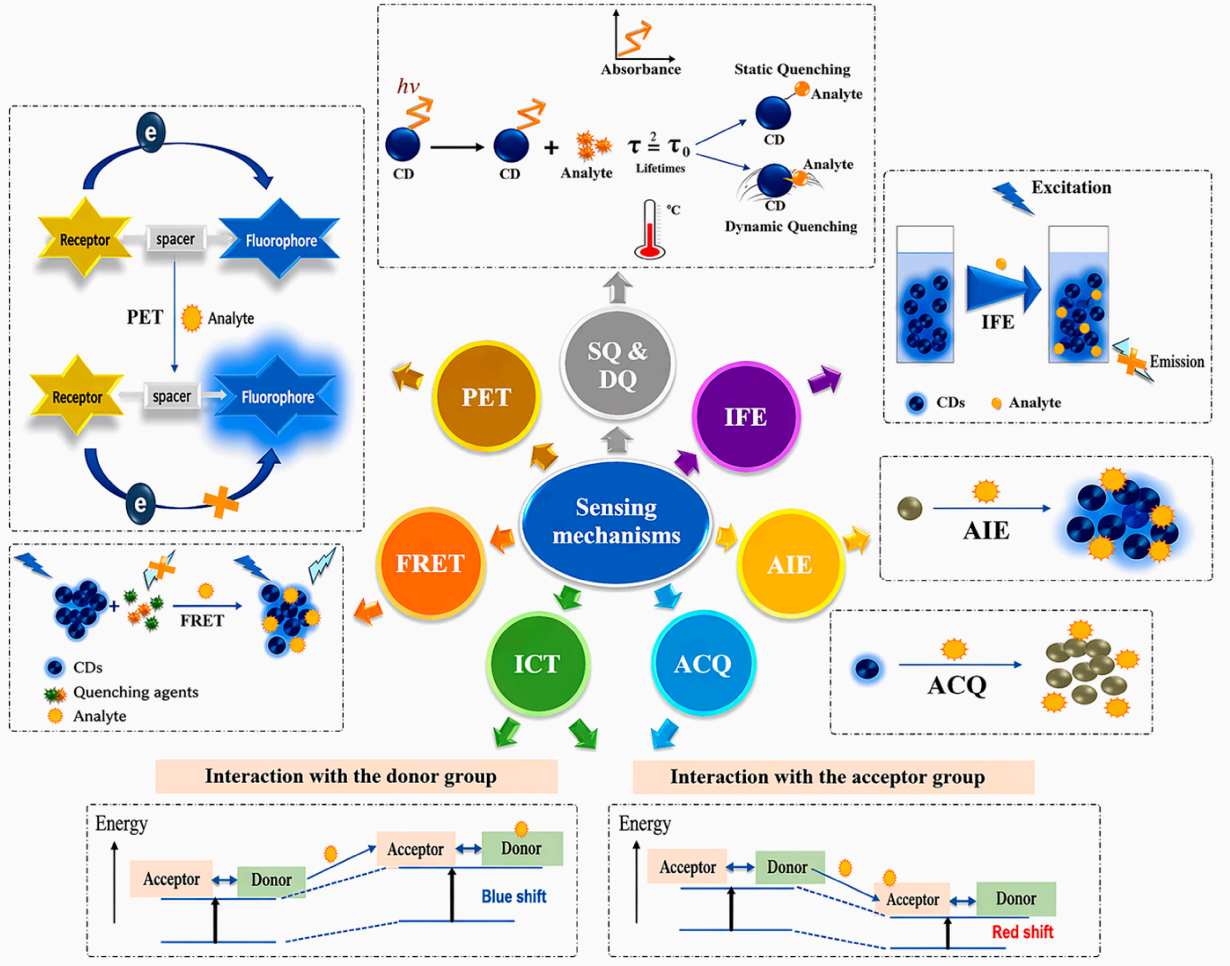
CDs induced possible photo-sensing mechanisms. Reproduced from [35] under CC BY 4.0 license.

The fluorescence properties of the CDs can be improved by doping N, S, P, and B, like heteroatoms, which can change the electronic states. In CDs, the fluorescence will originate through quantum confinement, surface defects, and molecular fluorophores. By proper functionalization and doping, multicolor stable fluorescent CDs can be fabricated. In general, the fluorescence quenching and enhancement paths are key in biosensing. The fluorescence on/off will work by adopting the following mechanisms. Such as photoinduced electron transfer (PET), Förster resonance energy transfer (FRET), inner filter effect (IFE), and static or dynamic quenching (SQ and DQ), aggregation caused quenching (ACQ), and aggregation-induced emission (AIE) [35,36]. Figure 4 shows various types of optical biosensing mechanisms.

#### 3.2.2. Electrochemical and Electron Transfer Properties

To be an efficient electrochemical biosensor, the electrode materials should possess good electron transfer capability [37]. Besides the metal-based nanomaterials, carbon nanomaterials have remarkable electrical conducting properties, high charge transfer kinetics, high surface area to accommodate many analytes, and recognizing molecules on their surface. The structural arrangement and sp^2^ hybridized carbon and CDs accelerate the electrical conductivity [38]. The CDs can act as transducers in biosensing technology to minimize the overpotentials and enhance the response to the analytes in lower concentrations of pharmaceutical, environmental, and other small molecules, as well as heavy toxic pollutants [39]. Conjugation of the CDs with other functional groups and doping can also accelerate the electron transfer, and target recognition by functionalizing with aptamers, enzymes, and antibodies [39,40].

#### 3.2.3. Surface Chemistry and Functional Groups

The functional groups on the CDs’ surface play a pivotal role in biosensor fabrication and analyte recognition by the biomolecules anchored at the end of the CDs [41,42]. A plethora of functional groups are present on top of CDs, such as carboxyl, hydroxyl, amine, and thiol [41,43]. These chemical groups impart hydrophilicity, water stability, and chemical reactivity to the nanomaterials and facilitate the bioconjugation [42,43]. The chemical structure of CDs allows the bioconjugation of additional functionalization through covalent and non-covalent interactions [41,42,43]. The versatility of carbon nanomaterials and CDs is the ease of surface functionalization and tunable surface chemistry, which can directly impact the sensitivity, operational stability, and response time of the CD-based biosensors [42,44,45]. The additional functionalization also has a remarkable impact on the luminescent and electrical properties of the CDs [41,44,45].

#### 3.2.4. Biocompatibility and Chemical Stability

Numerous cytotoxicity experiments have shown that CDs can be readily internalized into cells for imaging and have very minimal toxicity, whether they are surface passivated [46,47]. Tests of CDs’ cytotoxicity effects (viability, mortality, and proliferation) have been conducted using a variety of cell line types, concentrations, and surface coverages [46,47]. At concentrations adequate for cell labeling (about 10–100 μg mL^−1^), CDs have been shown to produce a minor reduction in cell viability in nearly every cytotoxicity study conducted to date [46,47,48].

UV light irradiation, temperature, salts (NaCl, KCl, etc.), pH, and other factors have been found to impact the properties of CQDs; therefore, the performance of CQDs should only be assessed in light of these aspects [49]. A material’s capacity to remain stable when exposed to radiation (UV, visible, etc.) is known as photostability. When exposed to light, fluorescent materials typically bleach over time. High photostability is therefore necessary in applications where materials are exposed to light for extended periods of time [49,50]. A material’s capacity to remain stable in a given heat environment is known as thermal stability. At high temperatures, the chemical structure of fluorescent materials typically changes, which causes the emission to decrease. For CDs to endure high-temperature applications, they must have good thermal photostability [49,50,51]. For CQDs to be used in real-world applications, ion, pH, or time stability is crucial. The ion stability was examined using the interference of specific common cations (at different concentrations) with the synthesized fluorescent CQDs. Similarly, the quenching of PL under various pH values was used to evaluate the pH stability. Time stability is a measure of how long the characteristics of CQDs are maintained. From an application standpoint, this is an extremely important characteristic because the time stability of CQDs will directly affect the lifespan of CQD-integrated systems [49,50,51].

**Figure 5:**
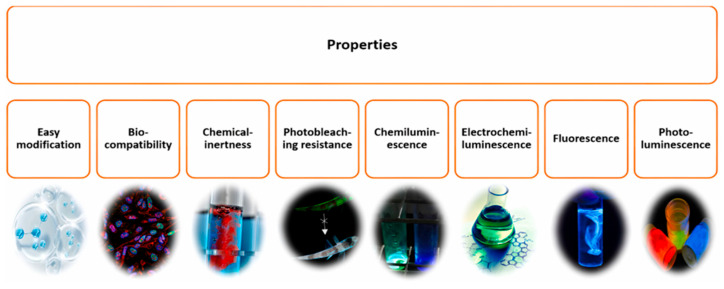
Various properties of CDs. Reproduced from [35] under CC BY 4.0 license.

Overall, CDs have excellent properties (see Figure 5) to stand as good candidates to serve as economic biocompatible biosensors to detect various contaminants.

## 4. Synthesis Strategies, Characterization, Scalability, and Reproducibility Considerations

**Figure 6:**
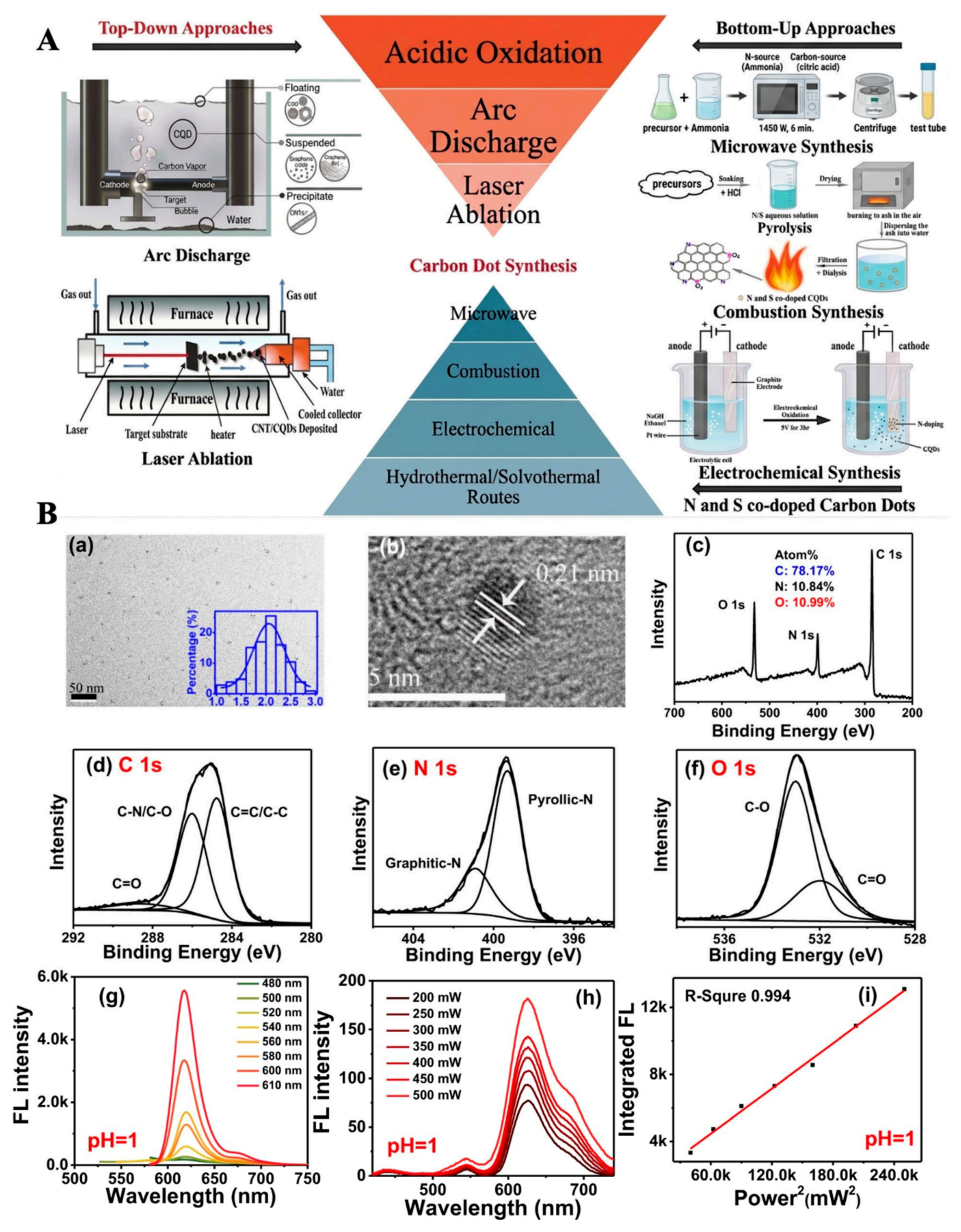
(**A**). Synthesis of CDs by top-down and bottom-up methods. Reproduced from [54] under CC BY 4.0 license. (**B**). (**a**). Transmission electron microscope (TEM) image of CDs with particle size distribution; (**b**). CD high-resolution TEM with 5 nm size scale bar; (**c**–**f**): CD full scan: C 1s, N 1s, and O 1s high-resolution XPS spectra; (**g**,**h**): one-photon and two-photon fluorescence (FL) spectra of protonated CDs in deionized water; (**i**): correlation between two-photon FL intensity and femtosecond (fs) laser power. Reproduced from [55] under CC BY 4.0 license.

There are many ways to prepare CDs, but they can be divided into two major groups according to how they are prepared: (1) top-down and (2) bottom-up methods [52,53]. To create CDs with nanoscale dimensions, the top-down method entails cutting or exfoliating bigger carbon-based materials. The raw materials are carbon powder, graphite rods, carbon nanotubes, and graphene, among others. To create carbon nanoparticles, the bottom-up approach assembles a huge number of small organic carbon atoms. The main sources of carbon are either organic molecules or small-molecule oligomers [29]. Arc-discharge, laser ablation, acidic oxidation, microwave pyrolysis, different combustion techniques, electrochemical synthesis, and hydrothermal or solvothermal processes are examples of top-down and bottom-up methodologies (See Figure 6A) [53,54]. The prepared CDs can be characterized using different spectroscopic and electron microscopy methods, as represented in Figure 6B [55].

CDs are gradually moving into industrial manufacturing due to the advent of scalable synthesis techniques, with many efforts concentrated on lowering equipment needs and improving process efficiency [56]. Hydrothermal, solvothermal, microwave, pyrolysis, and solid-state carbonization are only a few of the synthesis techniques that have been greatly improved to increase yield, lower energy usage, and achieve ecologically benign manufacturing. For example, the large-scale production of CDs for industrial use can be highly feasible by adopting microwave, pyrolysis, and solid-state carbonization, which is considered highly feasible because of simple operation and high output. Recently, great efforts have been made by various researchers in fine-tuning the optical properties and stability by incorporating various dopants and choosing the appropriate starting precursor [57,58,59].

Apart from these advancements in CD fabrication, many hurdles exist in real-time use and bulk production. Currently, at first, simple, stable, effective, economic, and eco-friendly industrial-scale production methods must be developed. Though hydrothermal and solvothermal methods are widely used, they need costly equipment, prolonged reaction times, and high energy consumption. Due to these reasons, the methods may not be suitable for continuous industrial production [57]. New approaches like solid-state carbonization [58] and CDs made from biomass have shown promise for lowering expenses and lessening their negative effects on the environment [60].

Second, in order to meet certain performance requirements, CDs must be precisely customized in a variety of application scenarios, from biomedicine and optoelectronics to smart packaging. For example, optoelectronics emphasizes optical tunability and quantum yield, whereas biomedical applications prioritize biocompatibility and stability [59,60]. However, a major problem continues to be striking a balance between the scalability and cost-effectiveness of synthesis and the growing complexity of multifunctional customization [11,33,61,62]. Third, there are several difficulties in converting laboratory-scale optimizations into industrial-scale procedures, especially when it comes to modifying synthesis methods for large-scale manufacturing. To guarantee consistency and scalability, this shift calls for advancements in process control, equipment design, and quality management. High-temperature and high-pressure circumstances are necessary for many traditional synthesis approaches, including hydrothermal and solvothermal processes. These temperatures not only make operations more complex, but they also place strict demands on industrial equipment. For CDs to be practically implemented in large-scale manufacturing, these technical requirements must be met [57,59,61].

## 5. Immobilization Strategies of Enzymes, Antibodies, and Aptamers on CDs

**Figure 7:**
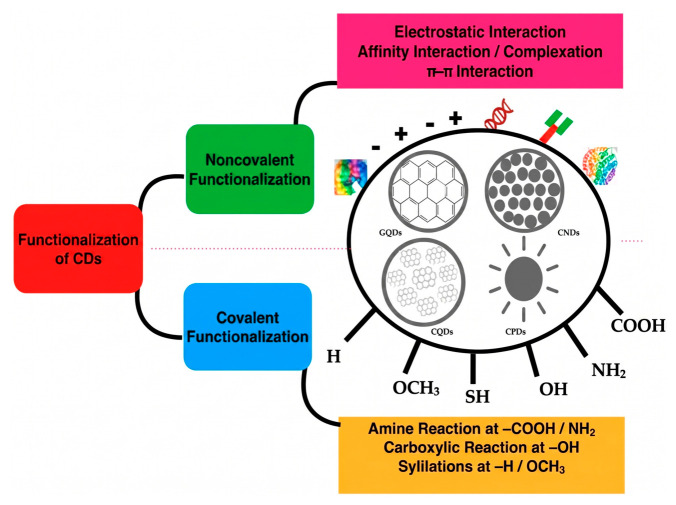
Surface functionalization of CDs with various biomolecules with covalent and noncovalent approaches.

Immobilization of biorecognition elements such as enzymes, antibodies, and aptamers onto CDs is a crucial step in the fabrication of efficient biosensors, as it directly affects sensitivity, selectivity, and operational stability of the sensing platform [63,64,65]. Chemical reports that the most commonly employed strategies include non-covalent and covalent interactions, as shown in Figure 7. The non-covalent interactions are physical adsorption, like π–π interactions, affinity interactions, or complexation, and electrostatic interactions. The covalent conjugation is an amine reaction, a carboxylic acid reaction, and silylations [66]. Physical adsorption exploits electrostatic forces, hydrogen bonding, and π–π stacking between biomolecules and surface functional groups of CDs, offering simple fabrication but relatively limited long-term stability and possible biomolecule leaching. Covalent immobilization, typically achieved via carbodiimide chemistry (EDC/NHS) between surface carboxyl groups of CDs and amino groups of enzymes or antibodies, ensures strong attachment, improved reproducibility, and higher resistance to environmental conditions. This approach is particularly advantageous for hybrid CD–metal or CD–polymer systems used in electrochemical and fluorescence biosensors [66]. In contrast, affinity-based immobilization strategies, such as biotin–streptavidin coupling, His-tag/metal coordination, and DNA hybridization, allow controlled orientation of antibodies and aptamers, thereby preserving biological activity and enhancing target recognition efficiency [67,68,69,70]. Overall, the choice of immobilization method depends on the required sensor stability, response time, and application domain, with covalent and affinity-based approaches being preferred for clinical and environmental monitoring systems.

## 6. Electrochemical CD-Based Nanobiosensors for Pharmaceutical Analysis

In this section, we will discuss CD-based biosensors and chemosensors for the analysis of pharmaceutical products.

Electrochemical sensing has emerged as one of the most powerful transduction strategies for CD-based biosensors because of its high sensitivity, operational simplicity, low cost, and compatibility with various platforms. In CD-modified systems, biochemical interactions occurring at the electrode interface are converted into measurable electrical signals such as current, potential, or impedance [24]. Compared to optical approaches, electrochemical sensors offer a faster response and are easily integrated into portable and wearable devices. Therefore, they are particularly suitable for pharmaceutical analysis and point-of-care (POC) applications [25].

Conventional electrodes, such as gold and bare carbon, possess good conductivity but exhibit limited sensitivity for detecting trace drug molecules. To overcome this limitation, CD and CD-based nanocomposites are introduced onto electrode surfaces, where they act as conductive nanostructures and functional scaffolds for the recognition of analytes [70]. The nano-sized CDs, along with their sp^2^-hybridized carbon frameworks, enable rapid electron transfer and substantially enhance electrochemical signals [71]. Moreover, CDs contain a lot of surface functional groups (such as–COOH, –OH, and –NH_2_), which facilitate strong adsorption of pharmaceutical compounds and allow covalent or non-covalent immobilization of biorecognition elements such as enzymes, antibodies, and aptamers [24]. This dual functionality—signal amplification and molecular recognition—forms the basis of CD-based electrochemical biosensing platforms.

### 6.1. CD–Modified Electrodes

#### 6.1.1. CDs in Electrode Surface Engineering

CDs enhance electrochemical sensing through several complementary mechanisms. Firstly, their large specific surface area increases the number of electroactive sites available for analyte interaction, leading to higher current responses [33]. Secondly, CDs reduce charge transfer resistance and overpotential, thereby improving their sensitivity towards redox-active drugs such as paracetamol and diclofenac [72]. Thirdly, surface functionalization allows selective binding of pharmaceutical molecules and biological receptors, thereby enabling targeted sensing in complex matrices such as serum or sweat [72].

#### 6.1.2. Hybrid Nanocomposite-Based CD Sensors for Pharmaceutical Analysis

Hybrid nanocomposites, formed by integrating CDs with metal nanoparticles or conducting polymers, have emerged as highly effective electrochemical sensing platforms for pharmaceutical analysis [73]. Since around 2020, these hybrid architectures have been increasingly investigated to overcome the limitations of single-component systems through the integration of the high conductivity, surface functionality, and biocompatibility of CDs with the catalytic or mechanical advantages of metals and polymers [74,75]. Such synergistic interactions result in improved electron transfer kinetics, enhanced sensitivity, and greater operational stability, particularly in complex pharmaceutical and biological matrices [76].

##### Metal–CD Hybrid Nanocomposites

Metal–CD nanocomposites combine the high electrocatalytic activity of metal nanoparticles with the electron transfer and adsorption properties of CDs, thereby creating efficient electrochemical sensing interfaces [77]. Gold–CD systems were among the earliest explored. Initially, they were employed to enhance electrode conductivity and stability; later, they enabled sensitive detection of pharmaceuticals such as ciprofloxacin, paracetamol, and dopamine with excellent analytical performance [76]. Silver–CD hybrids gained prominence after 2020 owing to the strong redox behavior and signal amplification of silver nanoparticles, supporting rapid and sensitive electrochemical detection of antibiotics, including sulfonamides and tetracyclines [66]. Platinum–CD nanomaterials emerged mainly after 2021 as high-performance catalytic sensing platforms, where platinum provides exceptional electrocatalytic efficiency while CDs improve nanoparticle dispersion and charge transport. These hybrids have been applied to detect anticancer drugs such as doxorubicin, cisplatin, and paclitaxel for therapeutic monitoring [78]. More recently, copper–CD nanocomposites have been investigated as cost-effective alternatives to noble metals, wherein CDs stabilize copper nanoparticles against oxidation and enhance conductivity, thereby enabling reliable electrochemical detection of antibiotics such as amoxicillin, ciprofloxacin, and metronidazole [67,68].

##### Polymer–CD Hybrid Nanocomposites

Since 2020, polymer–CD hybrid nanocomposites have attracted increasing attention due to their mechanical flexibility, film-forming ability, and chemical stability [72,79]. In these systems, conductive polymers form a continuous electron-transport network, while CDs enhance surface reactivity and facilitate interfacial electron transfer [80]. Polyaniline–CD (PANI–CD) composites were among the earliest polymer–CD hybrids used for pharmaceutical sensing. Incorporation of CDs (e.g., N-doped CQDs) into the PANI matrix reduces charge transfer resistance and improves conductivity and signal stability, enabling sensitive electrochemical detection of pharmaceutical drugs such as amoxicillin in drinking water and lake water [81]. Polypyrrole–CD (PPy–CD) composites became prominent after 2021 because of their strong redox activity and electrode adhesion, wherein CDs increase film porosity and conductivity, thereby accelerating electron transfer. These hybrids have been applied to detect antibiotics such as tetracycline, ciprofloxacin, and sulfamethoxazole, and have also shown amplified electrochemical and fluorescence responses for compounds like ampicillin and picric acid [82,83]. More recently, Poly (3,4-ethylenedioxythiophene)–CD (PEDOT–CD) nanocomposites have gained significant interest, particularly since 2022, for flexible and wearable sensing platforms. PEDOT offers high conductivity and mechanical durability, while CDs improve interfacial charge transport and analytical sensitivity [84]. PEDOT–CD sensors have been demonstrated for monitoring of pharmaceuticals such as paracetamol, caffeine, and antibiotics in sweat, saliva, and other biological fluids, enabling real-time drug analysis [85]. Systems based on PEDOT:PSS (PSS = Polystyrene sulfonate) combined with CDs or quantum dots have achieved nanomolar-level detection limits for antibiotics like amoxicillin. Moreover, optimized hybrid PEDOT films have enabled simultaneous detection of paracetamol and related analgesics, with some hybrid architectures (e.g., Au/PEDOT) achieving remarkably very low detection limits for drugs such as moxifloxacin [86].

#### 6.1.3. Standardization and Reproducibility of Hybrid CD–Metal Sensors

In industrial and medical applications, standardization and reproducibility remain major challenges in the preparation and application of hybrid CD–metal sensors. Significant batch-to-batch variations in precursor composition, synthesis routes, surface functionalization, and post-treatment procedures lead to inconsistent optical and electrochemical characteristics across laboratories. Jing et al. reported that the lack of reference standards and unified synthetic protocols significantly hinders the scalability and cross-comparability of CD-based systems, especially when intricate hybrid architectures are involved [84]. Similarly, Cayuela et al. emphasized that inconsistent reporting of particle size, surface chemistry, quantum yield, and stability parameters is a significant barrier to reproducible nanodot fabrication and performance benchmarking [87]. Furthermore, minor changes in CD core structure and surface states can have a significant impact on photoluminescence behavior and charge transfer efficiency, which in turn affects sensor sensitivity and reliability [88]. Therefore, standardized synthesis workflows, certified reference materials, and harmonized characterization protocols are crucial to guarantee repeatable performance, regulatory compliance, and large-scale manufacturability of hybrid CD–metal sensing platforms.

### 6.2. Electrochemical Sensing Modes

*Amperometric Sensing*: These sensors operate by measuring the current at a fixed potential during analyte oxidation or reduction. CD-modified electrodes typically exhibit faster electron transfer kinetics, resulting in higher sensitivity compared to unmodified electrodes [89]. This approach has been widely used for drugs such as paracetamol, wherein CD composites enable nanomolar-to-micromolar detection with minimal sample preparation [90].

*Voltammetric Techniques*: Cyclic voltammetry (CV) and differential pulse voltammetry (DPV) are extensively used for pharmaceutical analysis due to their ability to resolve multiple redox peaks simultaneously [91]. DPV, in particular, provides superior sensitivity and peak resolution, allowing multiplexed detection of drugs such as ibuprofen, diclofenac, and ciprofloxacin [92].

### 6.3. Functioning Mechanisms of Electrochemical CD Biosensor

**Figure 8:**
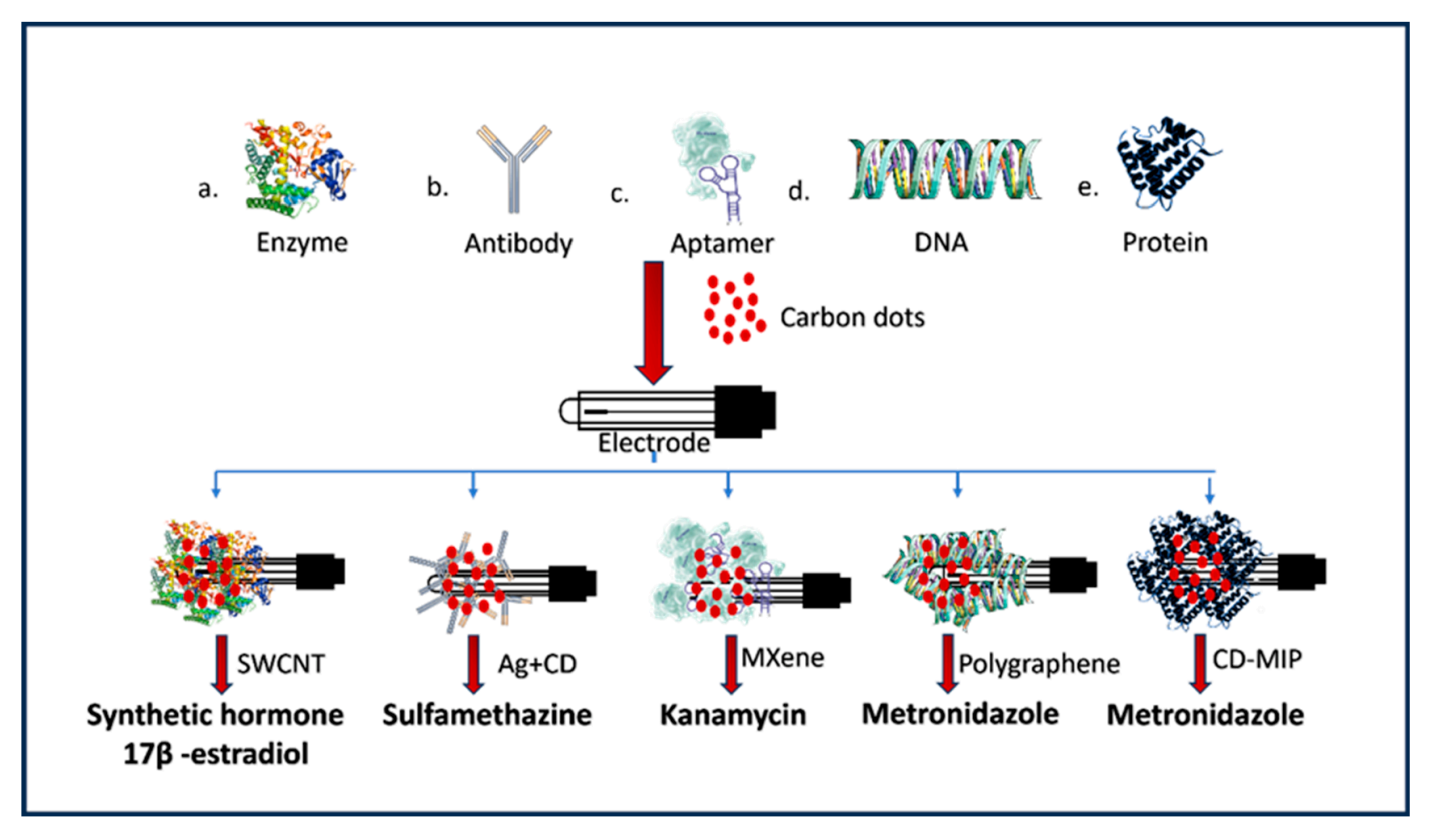
Schematic diagram of biomolecule-assisted CDs electrochemical nanobiosensors: (**a**) enzyme-based, (**b**) antibody-based, (**c**) aptamer-based, (**d**) DNA-based, and (**e**) protein-based.

Electrochemical CD biosensors function either through direct redox reactions of the analyte or via specific biological recognition mechanisms. Figure 8 showns various biomolecule-assisted nanobiosensors based on CDs.

#### 6.3.1. Enzyme-Based Systems

Enzyme–CD hybrids utilize enzymatic catalysis for pharmaceutical sensing, wherein CDs facilitate direct electron transfer and improve enzyme stability [93]. Canevari et al. fabricated a biosensor for the synthetic hormone 17α-ethynyl-estradiol using laccase immobilized on a hybrid of single-walled carbon nanotubes (SWCNTs) and CDs [94]. The incorporation of CDs significantly improved the electron transfer between the copper ion active sites of the laccase enzyme and the electrode surface, achieving a detection limit of 4.0 nmol. Baj-Rossi et al. utilized a cytochrome P450 (isoform CYP1A2)-modified electrode for the detection of naproxen [93]. The enzyme was immobilized on multi-walled carbon nanotubes (MWCNTs), which enhanced the bio-electrocatalytic activity, allowing for the continuous monitoring of the drug’s metabolism.

#### 6.3.2. Antibody-Based Sensor

CDs are widely employed in electrochemical immunosensors as nanocarriers and signal amplifiers. These systems are frequently applied in disease biomarker detection, particularly in cancer diagnostics [95]. In sandwich-type immunoassay configurations, CD-labeled antibodies enhance electron transfer and provide high antibody loading, thereby enabling sensitive pharmaceutical residues.

#### 6.3.3. Aptamer-Based CD Sensors

These sensors exploit the high specificity and affinity of nucleic acid aptamers toward pharmaceutical targets. CDs typically serve as conductive scaffolds or signal enhancers, while target binding induces conformational changes or probe displacement, resulting in measurable electrochemical responses [96,97]. Li et al. developed a self-assembly aptasensor for kanamycin detection using CD-decorated MXene. In this system, CDs are intercalated between the MXene layers, effectively preventing restacking and facilitating electron transfer. The CDs also provided abundant active sites for the immobilization of a double-stranded DNA (dsDNA) probe (aptamer hybridized with cDNA). Upon kanamycin binding, the aptamer released the cDNA and the methylene blue signal tag, leading to signal attenuation (a “signal-off” mechanism) [98]. Liu et al. and Mat Zaid et al. reported on 17β-estradiol detection [99]. Mat Zaid et al. constructed an impedimetric aptasensor by electrodepositing conductive CDs onto a screen-printed electrode. The binding of estradiol to the 76-mer aptamer hindered the redox probe’s access to the surface or altered the charge transfer resistance (R_ct_), allowing for picomolar detection limits (0.5 × 10^−12^ M) [99].

#### 6.3.4. DNA-Based CD Biosensors

DNA-modified CD electrodes have been applied to pharmaceuticals that interact with nucleic acids. The carcinogenic and mutagenic substances like nitrosamines can be detected by the DNA adhered electrodes as these analytes interact with DNA via intercalation or oxidative damage mechanisms, leading to measurable electrochemical signal changes [100]. Mahmoudi et al. designed a DNA biosensor based on a carbon-paste electrode-modified GQD for the detection of topotecan [101].

#### 6.3.5. Protein-Based Sensors

Non-enzymatic protein-assisted CD platforms mainly depend on affinity interactions or blocking proteins to improve selectivity. Kim et al. pioneered the use of avidin–biotin systems on electrode chips for estradiol sensing [102]. This affinity strategy has been adapted in CD-based sensors where CDs are functionalized with streptavidin/avidin to strongly bind biotinylated probes (aptamers or antibodies), ensuring stable immobilization and high selectivity [103]. Ensafi et al. and Li et al. explored molecularly imprinted polymer (MIP) composites for pharmaceutical targets [103,104]. Ensafi et al. synthesized CD-MIP nanocomposites for metronidazole detection using sol–gel transitions, while Li et al. used hollow MIPs on CDs for tetracycline. These “protein-templated” or protein-analogous cavities within the polymer-CD matrix allow for the highly selective recognition of specific drug molecules.

### 6.4. Applications in Pharmaceutical Analysis

#### 6.4.1. Detection of Active Pharmaceutical Ingredients (APIs)

Electrochemical CD-based sensors have been used to detect a wide range of APIs (see Table 1). Their success stems from two routes: (i) direct electrocatalytic oxidation/reduction of the drug at a CD-enhanced electrode, and (ii) indirect detection using CDs as signal amplifiers in conjunction with selective recognition elements (aptamers/antibodies) or catalytic additives [105,106].

*Paracetamol (acetaminophen)*: Paracetamol is a classic target for CD-electrochemical sensors because it shows a clear, well-defined oxidation peak in the graph. Strategies that combine CDs with conducting polymers such as polyaniline or PEDOT: PSS also improve film stability on flexible electrodes for repeated measurements [107].

*Ciprofloxacin and other antibiotics*: Ciprofloxacin has been detected using hierarchical carbon/NiCo composites and ZnO–CD hybrids, which provide both electrocatalytic activity (metal oxide) and conductive pathways (CDs) [108]. These hybrids give better selectivity in complex matrices (urine, river water, and formulation samples) and often achieve LODs in the low µM range; fluorescence-based CD probes have demonstrated nM sensitivity and validate the high intrinsic sensitivity of CD cores when appropriately engineered [109]. Metal–CD and metal oxide CD hybrids, such as ZnO@CD and AgNP@CD, are particularly useful when the antibiotic interacts with metal-mediated redox chemistry [110,111].

*Non-steroidal anti-inflammatory drugs (NSAIDs) and analgesics*: Ibuprofen, diclofenac and related NSAIDs are typically less electroactive than paracetamol, so hybrid strategies have been effective: CDs combined with graphene or MWCNTs increase the electroactive area and improve peak separation for simultaneous determinations in multi-component formulations [112]. For example, fullerene–carbon nanofiber and graphene–carbon nanotube paste electrodes have been successfully employed to distinguish the oxidation potentials of these co-existing drugs in water samples [113].

*Anticancer and antiviral drugs*: For challenging molecules such as fluorinated anticancer drugs, CD hybrids with noble metal nanoparticles (Au and Pt) and MOFs have been used to obtain catalytic amplification that resolves overlapping peaks and lowers LODs [114,115]. Aptamer-functionalized CD layers further increase specificity in biologically relevant media, as seen in chemiluminescent and electrochemical platforms designed for high-precision drug monitoring [116].

**Table 1:** Carbon dot-based electrochemical, fluorescence, and optical sensing platforms for pharmaceutical analyte detection.

Pharmaceutical Analyte	Detection Technique	Detection Limit	Type of Electrochemical Biosensor	Ref. No.
17β-Estradiol	Electrochemical impedance spectroscopy	Picomolar range	Aptamer-functionalized carbon dot modified electrode	[100]
Amoxicillin	Amperometric detection	~0.03 µM	Polyaniline/AgBr combined with CDs	[81]
Caffeine	Differential pulse voltammetry	Low µM range	Carbon dot–chitosan composite modified electrode	[117]
Ciprofloxacin	Cyclic voltammetry (electrochemical mode of dual-mode platform)	~0.082 µM	Label-free bio-derived carbon dot modified electrode	[118]
Doxorubicin	Cyclic voltammetry	~0.09 µM	Screen-printed carbon electrode modified with carbon dot–magnesium oxide nanocomposite	[119]
Doxorubicin	Voltammetry	Low µM/sub-µM	Carbon dot–cerium oxide modified screen-printed electrode	[120]
Metronidazole	Differential pulse voltammetry	~0.18 µM	Carbon dot–metal oxide composite non-enzymatic electrochemical sensor	[121]
Ofloxacin	Differential pulse voltammetry (dual mode)	~0.127 µM	Biomass-derived carbon quantum dot electrochemical/fluorescence sensor	[122]
p-Aminophenol (paracetamol impurity)	Differential pulse voltammetry	0.0456 µM	Same nitrogen-doped carbon dot/manganese oxide hybrid electrode	[123]
Paracetamol	Differential pulse voltammetry	0.0303 µM	Glassy carbon electrode modified with nitrogen-doped CDs decorated with manganese oxide nanospheres	[123]
Tetracycline	Differential pulse voltammetry	~0.15 µM	Polypyrrole–carbon dot composite electrochemical biosensor	[109]
Theophylline	Differential pulse voltammetry	Low micromolar range	Carbon dot–polymer composite electrochemical sensor	[120]
Chloramphenicol	Fluorescence quenching	0.12 µM	Red-emissive CDs	[124]
Ciprofloxacin	Turn-off fluorescence (dual-mode platform)	~0.293 µM	Bio-derived carbon dot fluorescence/electrochemical dual sensor	[125]
Kanamycin	Fluorescence recovery aptasensor	0.09 µM	Carbon dot–aptamer fluorescence probe	[126]
Ofloxacin	Fluorescence quenching (dual-mode)	~0.127 µM	Rice-husk-derived carbon quantum dot dual-mode sensor	[127]
Oxytetracycline	Fluorescence turn-off	0.374 µM	Carbon quantum dot fluorescent probe	[128]
Sulfamethazine	Fluorescence quenching	0.18 µM	Carbon quantum dot optical probe	[129]
Tetracycline	Fluorescence quenching	0.236 µM	Nitrogen-doped carbon quantum dots	[109]
Chloramphenicol	Colorimetric/fluorescence	0.095 µM	Carbon dot colorimetric probe	[130]
Ofloxacin	Fluorescence + electrochemical dual mode	~0.127 µM	Biomass carbon quantum dot optical/electrochemical sensor	[127]
Tetracycline	Fluorescence–colorimetric dual sensing	0.14 µM	Carbon dot–metal ion optical probe	[131]

#### 6.4.2. Therapeutic Drug Monitoring (TDM) and Biological Matrices

TDM in blood, saliva, and sweat-TDM demands accurate, interference-resistant quantification in complex biological fluids. CD-based sensors help in three ways: (i) surface functionalization (e.g., with specific peptides or functional groups) reduces non-specific adsorption and improves binding affinity; (ii) hybrid composites, such as those combining conducting polymers and metallic nanoparticles, increase the signal-to-noise ratio by enhancing electron transfer pathways; and (iii) aptamer/antibody immobilization provides the necessary selectivity for clinical diagnostics [35,132].

*Blood*/*plasma*: For high-precision TDM, CD+ AuNP and CD+ graphene hybrids with antibody or aptamer capture have given promising results for anticancer drugs and narrow-window therapeutics. For example, a green-synthesized CD-modified electrode was successfully used to detect paclitaxel in human serum and urine with acceptable reproducibility (RSD = 2.6%) and recovery rates (97.7–103.0%), showing good resistance to common interfering species like dopamine and glucose. Additionally, bio-functionalized CDs conjugated with antibodies have been designed for the ultrasensitive detection of biomarkers in blood serum, achieving significantly higher sensitivity compared to conventional immunoassay methods [95,132,133].

Saliva/sweat: Non-invasive media are ideal for wearables. CDs integrated into flexible polymer matrices (e.g., PANI and PEDOT: PSS) on screen-printed electrodes (SPEs) have been used to measure metabolites in sweat and saliva with reasonable sensitivity due to intimate skin contact and continuous sampling. A notable example includes a flexible carbon nitride quantum dot (CNQD)/PANI nanocomposite sensor capable of monitoring glucose in sweat; the pyridinic nitrogen in the CDs improved charge mobility in neutral pH environments (typical of sweat), preventing the cracking often seen in rigid electrocatalytic layers during movement [134,135].

#### 6.4.3. Wearable Platforms and Data Integration

Wearable CD sensors combine thin-film CD composites (CD+ conducting polymer or CD+ graphene) with flexible electronics and wireless telemetry. Key examples in open access literature include patches and wristbands integrating CD-polymer films for continuous monitoring and onboard amperometric readout [136]. The integration of these sensors with cloud analytics and Artificial Intelligence (AI) is an emerging trend, where AI-assisted data processing helps interpret complex signals, reduce noise, and potentially provide personalized dosing recommendations by analyzing real-time data trends [137].

Challenges include biofouling, calibration drift, and skin-matrix variability. To mitigate these issues, composite strategies such as using polymeric coatings (e.g., hyper-branched polymers or specific anti-fouling layers) and bio-compatible surface modifications have been explored to prevent protein adsorption and ensure signal stability over time [137,138].

*Wearable, POC and Future Directions*: Design considerations for POC and wearable CD sensors: Device reliability depends on material selection (stable CDs and adhesion layers), power management (low-power amperometry or self-powered biofuel cells) [136], and reproducibility (standardized CD synthesis). Hybrid choices vary by use case:

- *High-stability POC readers*: composites on glassy carbon electrodes (GCEs) are preferred for clinic-grade sensitivity and have been applied to the detection of cancer biomarkers and drugs with picomolar detection limits [25,139]. Smartphone-based readouts coupled with these materials are also being developed to lower instrumental costs [140].
- *Disposable wearables*: CD+ polymer (e.g., PANI and PEDOT:PSS) stacks on flexible substrates like polyethylene terephthalate (PET) or paper are ideal for low-cost and flexible applications, retaining high sensitivity even after mechanical bending tests [141].
- *Environmental robustness*: CD+ metal oxide (e.g., ZnO, TiO_2_, or Mn-Fe hybrids) layers are utilized for their oxidative stability and ability to function in variable pH and ionic strength environments, often required for environmental or field screening [142,143].

### 6.5. Regulatory, Scale-Up and Reproducibility Hurdles

For clinical translation, reproducible large-scale CD synthesis and quality control of the products are crucial. Green and microwave/solid-state carbonization methods have shown promise for scalability and low-cost production [144]. Standardization across batches produced on a large scale remains a good challenge. Impurities and aggregates from synthesis can alter physicochemical properties, affecting sensor performance [145]. Furthermore, clinical deployment faces strict regulatory hurdles regarding biocompatibility and manufacturing consistency; sensors must demonstrate non-toxicity and uniform performance to meet safety and efficacy standards required by bodies like the FDA [146]. Consequently, the literature calls for rigorous interlaboratory validation, the use of standard reference materials, and consensus on reporting protocols for LOD, linear range, and long-term stability testing.

### 6.6. Future Opportunities: Multiplexing, AI, and Hybrid Materials

Multiplexed arrays of CD-modified microelectrodes (each with a specific aptamer or MIP) enable simultaneous detection of parent drugs and metabolites; integrated with AI, these arrays can provide real-time pharmacokinetic profiles [147]. Emerging material combinations that deserve attention include CD + MXene for ultra-fast electron transfer, CD + MOF for molecular sieving and preconcentration, and CD + biomimetic polymers for anti-fouling TDM interfaces [148,149].

## 7. Optical CD-Based Nanobiosensors

### 7.1. Fluorescence-Based CD Sensors

#### 7.1.1. Mechanisms: Turn-On/Turn-Off and Ratiometric Sensing

Fluorescence-based sensors represent the most extensively studied class of carbon dot sensors. These sensors operate based on changes in the fluorescence emission of CDs in response to the presence of specific analytes [150]. Fluorescence-based CD sensors are among the most widely explored optical nanobiosensors due to their high sensitivity, low cost, simple operation, and excellent photophysical properties, including tunable emission, strong photostability, and biocompatibility [151]. Cryptically, fluorescence sensors operate by monitoring changes in the emission intensity or wavelength of the CDs when they interact with target analytes—e.g., drugs, ions, biomolecules—making them powerful tools for pharmaceutical analysis.

#### 7.1.2. Fundamental Principles of Fluorescence-Based Sensing

When excited by UV or visible light, CDs emit fluorescence whose intensity and wavelength distribution depend sensitively on their environment. Sensor designs exploit this sensitivity to report the presence and concentration of analytes by changes in fluorescence [152].

The two well-known primary types of fluorescence mechanisms are as follows.

Turn-on fluorescence: The fluorescence emission of carbon dots is activated when an analyte is introduced. For example, the fluorescence intensity of carbon dots increases upon binding to a target molecule like metal ions or biomolecules. A turn-on fluorescence reagent is an excellent phosphor that has a structural “defect,” such as an open cycle, a break in a conjugated bond system, or an attached quencher, that reduces the quantum yield. An extremely luminous product is produced when the analyte reacts with the error, deleting it. The proposed classification for substances with turn-on fluorescence depends on the types of defects and the reactions for their “correction.” In contrast, some sensors show enhanced fluorescence when binding analytes disrupt quenching interactions or induce structural changes that favor radiative emission. These turn-on behaviors provide high contrast and lower background noise. The “off–on–off” sensing strategies, which combine quenching and recovery steps, allow sophisticated control and detection in complex samples [34,35].

Turn-off fluorescence: In this mechanism, the fluorescence of carbon dots is quenched upon interaction with a target analyte. This quenching effect is often used to indicate the presence of specific drugs, metal ions, or other molecular species. Many CD sensors rely on fluorescence quenching when analytes interact with the carbon dots. Quenching can occur through mechanisms like electron transfer or the IFE, where the presence of an analyte absorbs excitation/emission light, diminishing fluorescence [34,35].

**Figure 9:**
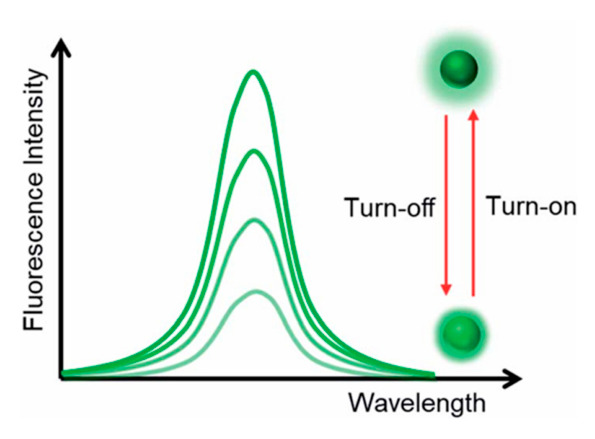
An illustration of fluorescence quenching (“turn-off”) and enhancement (“turn-on”). Reproduced from [154] under CC BY 4.0 license.

For example, many small molecules (like nitroaromatic compounds or antibiotics) can reduce CD emission via IFE-mediated quenching [153]. As generally shown in Figure 9, the “turn-off/on” mechanism depends on alterations in the overall fluorescence intensity based on emission quenching (“turn off”) or enhancement (“turn on”) [154].

When an analyte is present in fluorescence “turn-off” sensors, the emission of the fluorophore or phosphor is partially or completely quenched. Most Upconversion nanoparticle-based “turn-off” sensors are built using a method that quenches the emissions of Upconversion nanoparticles when an analyte is present, causing energy transfer processes to occur. Upconversion nanoparticles have been utilized in conjunction with AuNPs, dyes, magnetic nanoparticles, and graphene quantum dots, among other materials, to create “turn-off” platforms for a variety of uses, including virus detection and pesticides [154]. Ratiometric sensing: This method measures changes in the intensity ratio between two different emission wavelengths. This allows for more reliable detection, as the ratio is less sensitive to environmental conditions like pH or temperature fluctuations.

Unlike single-signal sensors, ratiometric methods measure the ratio of two emission intensities (often from dual emission peaks or combined fluorophores). Because the ratio is internally self-referencing, this method reduces errors due to instrument drift, environmental conditions, and sample variability—a major advantage in real-world pharmaceutical samples [155].

For instance, CDs can be designed with two emissive centers or combined with other fluorophores to provide dual intensity signals whose ratio changes upon analyte binding. Ratiometric detection is particularly useful for quantitative analysis and visual perception of concentration changes.

**Figure 10:**
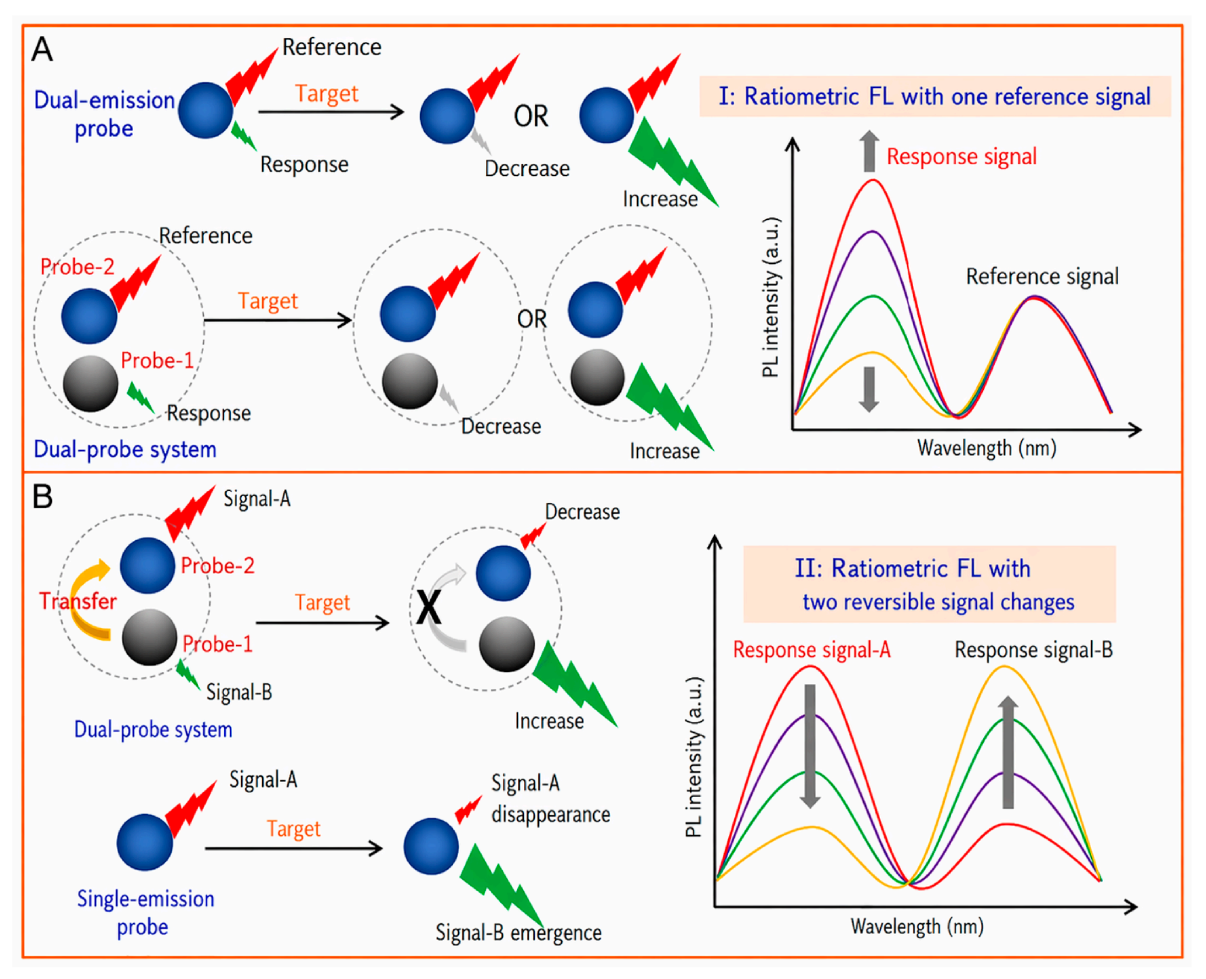
(**A**). The broad classifications to produce dual-emission ratiometric fluorescent sensors. (**B**). Ratiometric fluorescence using either two reversible signal changes or a single reference signal. Reproduced from [155] under CC BY 4.0 license.

The reference signal and the analyte-sensitive signal come from two separate probes, as seen in Figure 10A. The latter makes it possible to normalize the former. Basically, physically combining the two probes is a simple method of achieving this ratiometric biosensing or cell imaging. Nevertheless, the need for two separate probes may complicate the procedures. False imaging results, for instance, can be caused by unequal distributions of these two probes in cells. One benefit of single nanoprobes with dual-emission signals is that mistakes caused by changes in probe concentration are eliminated [155]. Using a single nanoprobe to produce dual-emission signals requires preconjugation or preassembly. There are two ways to accomplish this: chemical and physical. This design approach increases the single nanoprobes’ dependability and encourages their use in cell imaging and biosensing.

Figure 10B shows another method for creating ratiometric dual-emission sensors. There are reversible changes in two related analyte-sensitive signals. Generally speaking, when analytes are present, one signal may rise while another falls. The ratio of the two fluorescence signals has clearly changed. Building nanoprobes with two signal outputs that can cause analyte-binding-driven emission events, such as proton transfer, charge transfer, energy transfer, chemical reaction, or physical interactions, is a popular strategy for this ratiometry. Ratiometric fluorescence detection is made possible by the system’s ability to produce reversible variations in two signals through the particular interaction of probes with analytes [155].

**Figure 11:**
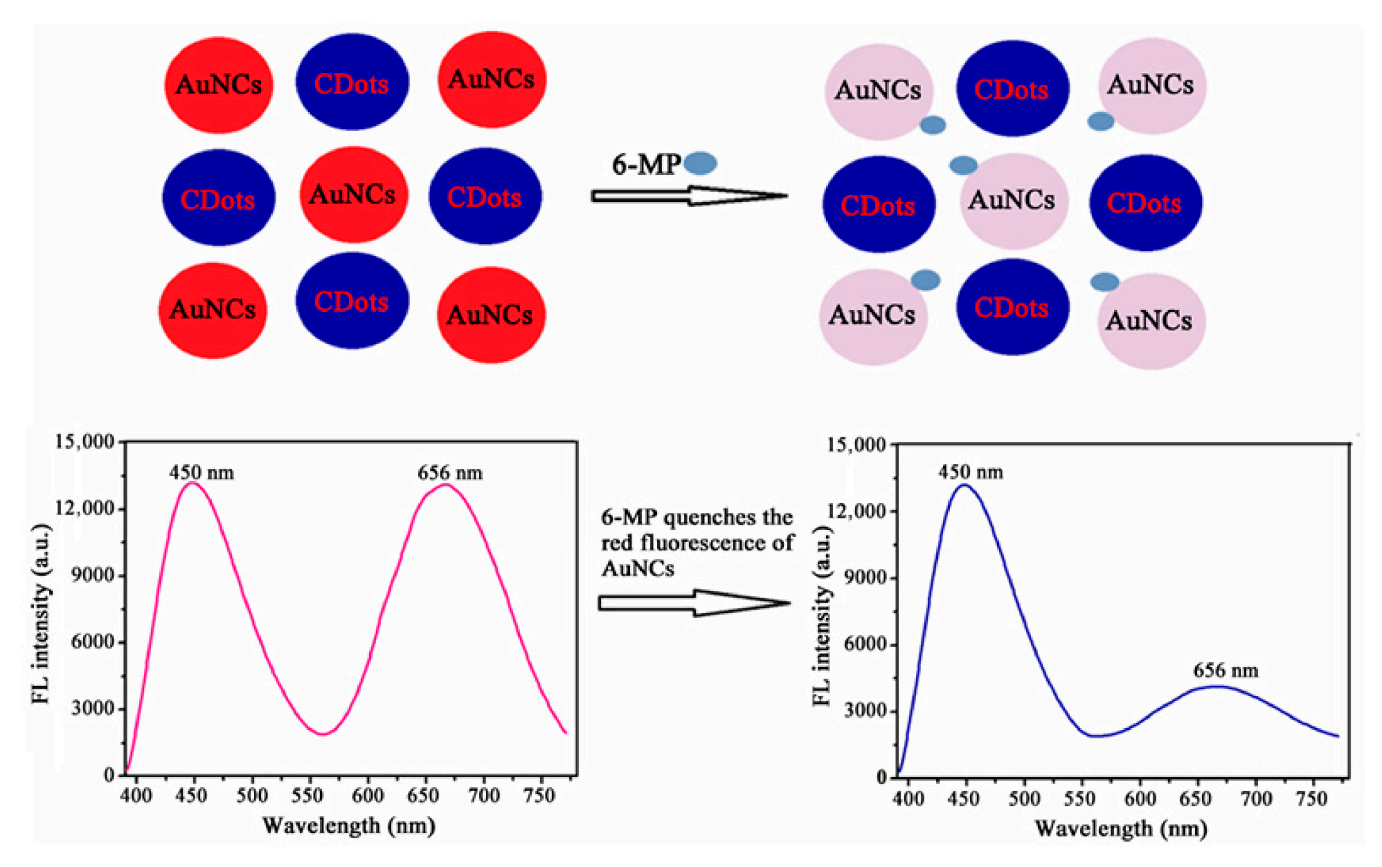
The detection principle of 6-MP is illustrated schematically: the red fluorescence emitted by AuNCs is suppressed upon interaction with 6-MP, whereas the blue fluorescence from CDs remains unaffected, providing a stable reference signal. Reproduced from [156] under CC BY 4.0 license.

Figure 11 presents a schematic of the ratiometric fluorescence bioassay platform designed for 6-MP (6-mercaptopurine) detection using a nanohybrid composed of CDs (CDots) and gold nanoclusters (AuNCs). Initially, CDs and AuNCs were synthesized. When 6-MP is introduced, its sulfhydryl group preferentially binds to AuNCs through a thiol–Au interaction, leading to significant quenching of the AuNC fluorescence. In contrast, the emission of CDs remains stable, serving as a reliable internal reference. By integrating these two fluorescence responses, the nanohybrid system provides an effective ratiometric approach for quantifying 6-MP, with AuNCs functioning as the recognition element and CDs acting as the reference fluorophore [156].

### 7.2. FRET-Based Sensing Strategies

FRET is used in conjunction with carbon dots to detect molecular interactions. The energy from an excited donor (carbon dot) is transferred to an acceptor molecule when in close proximity, resulting in a measurable change in fluorescence [157]. This technique enhances the sensitivity and selectivity of the sensor. FRET-based sensing strategies exploit nanoscale energy transfer between fluorophores to detect molecular events. By translating binding, cleavage, or conformational changes into fluorescence signals, these sensors provide powerful tools for studying biological and chemical processes with exceptional sensitivity and spatial precision.

FRET-based sensing strategies use the proximity-dependent energy transfer between a donor and acceptor fluorophore to detect analytes or monitor biological processes, relying on changes in distance (typically <10 nm) or environment to alter the signal, with common approaches including molecular beacons for DNA, logic gates for complex analysis, and using nanoparticles/quantum dots for enhanced signal, enabling sensitive, specific detection of ions, proteins, DNA, and conformational changes in real-time, without needing direct biomolecule labeling [158].

In FRET-based sensors, energy transfer occurs non-radiatively from a fluorescent donor (e.g., CD) to an acceptor when they are in close proximity (<10 nm) and the donor emission overlaps the acceptor absorption spectrum [155,157]. This coupling results in quenching of donor emission and enhancement (or altered emission) of acceptor signal.

This mechanism enables high selectivity and sensitivity for analytes that disrupt or enable the FRET pathway, such as drugs or biomolecules.

#### 7.2.1. Distance-Based (Conformational Change) FRET Sensors

Distance-based FRET sensors, also called conformational change FRET sensors, are among the most widely used FRET-based sensing strategies. They rely on the principle that small structural rearrangements within a biomolecule can produce large changes in FRET efficiency, due to the strong distance dependence of Förster energy transfer [159,160,161].

FRET efficiency depends on the donor–acceptor distance (1/r^6^ relationship). The binding of a target molecule causes a structural rearrangement of the sensor. This rearrangement changes the donor–acceptor spacing, producing a measurable fluorescence signal change [159,160,161]. A typical distance-based FRET sensor consists of a donor fluorophore (e.g., CFP), acceptor fluorophore (e.g., YFP), sensing domain (binds analyte), and flexible linker (allows conformational movement). These components are commonly engineered into a single fusion protein. Examples are (i) calcium sensors (Cameleon)—calmodulin-based, used for Ca^2+^ imaging; (ii) kinase activity sensors—phosphorylation-induced conformational change; (iii) metabolite sensors—ATP, glucose, and cAMP sensors. These sensors have high sensitivity, specificity, and suitability for live-cell imaging and make them powerful tools in biological and biomedical research [160].

#### 7.2.2. Binding-Induced FRET Sensors

Binding-induced FRET sensors are FRET-based sensing systems in which the donor and acceptor fluorophores are attached to separate molecules, and FRET occurs only when specific binding or association brings the fluorophores into close proximity (1–10 nm). FRET efficiency depends on the distance and orientation between donor and acceptor fluorophores.

In the unbound state, fluorophores are far apart, i.e., no or low FRET [161]. Upon specific molecular binding, donor and acceptor come close, and finally, the FRET signal appears or increases. The change in fluorescence intensity or ratio indicates the binding event in this particular binding strategy of the FRET sensors. Binding-induced FRET sensors consist of a donor-labeled molecule (protein, DNA, ligand), an acceptor-labeled molecule (binding partner), and a target analyte that promotes molecular interaction. The donor and acceptor are on different molecules, unlike conformational change sensors.

In this strategy, the donor fluorophore is excited by light. In the absence of binding, the donor emits fluorescence; no FRET signal occurs, and the binding event brings the acceptor close to the donor, while energy transfer ensues, and donor emission decreases, acceptor emission increases. Thus, their ability to directly report binding interactions makes them valuable tools in biochemical analysis, diagnostics, and molecular biology [162].

#### 7.2.3. Enzyme Activity-Based (Cleavage) Sensors

Enzyme activity-based (cleavage) sensors are biosensors that detect the presence or activity of an enzyme by monitoring the cleavage of a specific substrate. The sensor that does not just detect the enzyme—it detects what the enzyme does. A specific substrate is attached to a reporter system (e.g., fluorophore, chromophore, and electrode surface). The target enzyme recognizes and cleaves the substrate. This cleavage causes a measurable signal change, such as an increase/decrease in fluorescence, a change in color, a change in electrical signal, and the signal intensity is proportional to enzyme activity [161].

This sensor contains the substrate peptide/molecule (enzyme-specific), signal reporter, fluorophore, quencher, chromophore, electrochemical tag, transducer, i.e., optical, electrochemical, colorimetric factors. Based on these factors, enzyme activity-based (cleavage) sensors detect enzymes by measuring the signal produced when a specific substrate is cleaved by the target enzyme [162].

**Figure 12:**
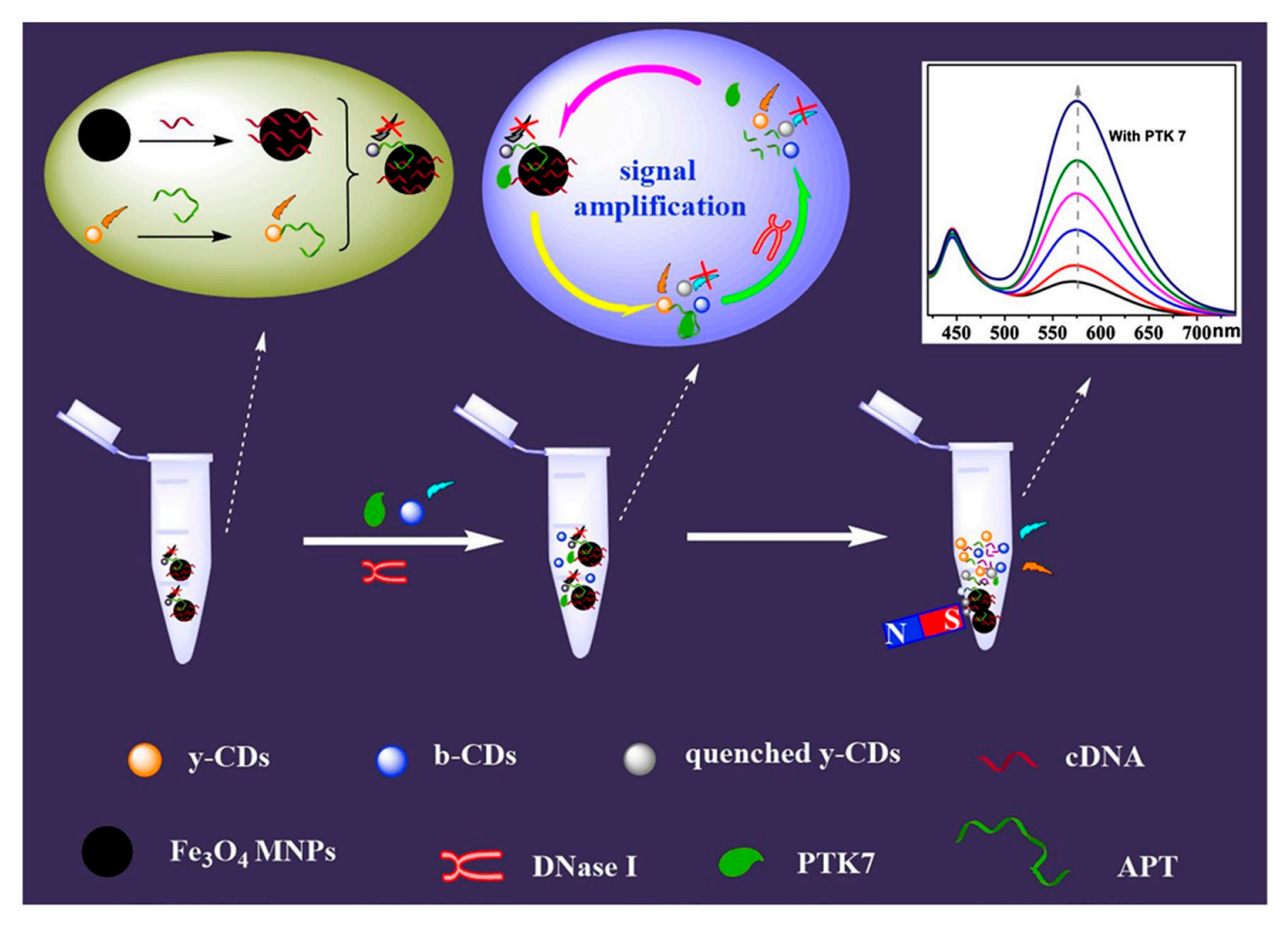
Schematic diagram related to the detection mechanism of the PTK 7 in a ratiometric fluorescence probe. Reproduced from [163] under CC BY 4.0 license.

A ratiometric fluorescent probe based on dual CDs was reported to detect PTK 7, and its efficacy was tested on actual samples. Figure 12 describes this ratiometric fluorescent probe’s manufacturing procedure and PTK7 detection method. First, APT (the PTK7 aptamer) was conjugated with y-CDs to generate y-CDs-APT, then cDNA (complementary to a portion of the PTK7 aptamer) was joined with Fe_3_O_4_ to form Fe_3_O_4_-cDNA. The unique bond between Fe_3_O_4_-cDNA and y-CDs-APT (y-CDs-APT cDNA- Fe_3_O_4_) suppressed the fluorescence of y-CDs. After PTK7 was added, y-CD fluorescence was recovered as y-CDs-APT coupled with PTK7 (y-CDs-APT-PTK7) to separate it from Fe_3_O_4_-cDNA. After that, DNase I was added to cleave the APT of y-CDs-APT-PTK7, releasing PTK7. The free PTK7 then broke the y-CDs-APT-cDNA-Fe_3_O_4_ once more, and more y-CDs were extracted from the Fe_3_O_4_ surface, and a loop amplifier was created. In summary, the detection signal of this ratiometric fluorescence probe was provided by y-CDs and b-CDs, and was amplified by DNase Ι. Several study articles have been examined to identify a potential detection mechanism for this probe. The fluorescence of y-CDs was clearly muted by the inner-filter effect based on the considerable UV absorption (Figure 12) of Fe_3_O_4_ MNPs [163]. However, y-CDs with higher concentrations quenched the fluorescence of b-CDs at a specific concentration, and the degree of quenching increased. Furthermore, the UV–vis absorption spectrum of y-CDs and the fluorescence spectrum of b-CDs were shown to significantly overlap. It suggests that an inner filter effect or fluorescence resonance energy transfer between b-CDs and y-CDs could be the source of the quenching [164].

Nevertheless, using the probe to identify PTK 7 did not extinguish the fluorescence of b-CDs. This research group examined the fluorescence lifespan spectra of b-CDs, b-CDs + y-CDs, and b-CDs + y-CDs-APT to determine the internal cause of whether the fluorescence of b-CDs was quenched, but they found no change after mixing with y-CDs-APT. The fluorescence lifetime of b-CDs, meanwhile, was lowered if there was an energy transfer [163]. The APT-modified y-CDs increased the molecular gap between y-CDs and b-CDs, hence destroying their electron or energy transfer mechanism. All the above findings show that the probe’s detecting mechanism does not include any energy or photoelectron transfer processes, which implies that the sensor operates in a more regulated manner.

### 7.3. CDs-Assisted Raman and Surface-Enhanced Raman Scattering (SERS) Platforms

The carbon dot-assisted Raman platform is a Raman sensing system in which CDs are used to enhance, modify, or improve Raman signals by increasing analyte adsorption, signal stability, or chemical enhancement, thereby improving sensitivity and detection efficiency of Raman spectroscopy [165]. Raman spectroscopy is a powerful analytical technique used in pharmaceutical analysis for drug identification, polymorphism studies, impurity detection, quality control, and many other applications. However, normal Raman signals are weak. To overcome this, various CDs with different functions are used as signal-enhancing and signal-modifying nanomaterials, leading to the CD-assisted Raman platform [166]. The CD-assisted Raman platform is a promising, cost-effective, and biocompatible approach for enhancing Raman signals in advanced pharmaceutical analysis, especially for low-concentration drugs, impurities, and quality control applications. The comparative table of the normal Raman [167] and the CD-assisted Raman sensing properties is displayed in Table 2. CDs are mixed with a low-concentration drug sample. The drug molecules adsorb onto the surface of carbon dots, and due to charge transfer and molecular enrichment effects, the Raman signal intensity increases, allowing sensitive detection.

SERS in sensors is a highly sensitive sensing system that uses specially designed metal nanostructures (usually silver, gold, or copper) to enormously amplify Raman signals of molecules, enabling ultra-trace and even single-molecule detection. In other words, A SERS platform is a sensor that uses metal nanoparticles to make very weak Raman signals extremely strong for detecting tiny amounts of substances [166,167].

#### 7.3.1. Signal Enhancement Mechanisms

Raman platforms mainly use chemical and resonance enhancement mechanisms, whereas SERS platforms achieve ultra-high sensitivity mainly through electromagnetic plasmonic enhancement along with chemical enhancement [168].

#### 7.3.2. Pharmaceutical Sensing Applications

Recently, Yanqiu Yang et al. reported [169] the novel Ag-CDs-PBA (phenyl boric acid) nanocomposites to detect toxic malachite green (MG) molecules with high SERS sensitivity and good uniformity with rich binding surface sites. Colorimetric sensors based on carbon dots rely on changes in the optical properties, particularly the color of the solution, upon exposure to a target analyte. These changes arise due to aggregation, surface functionalization, or specific binding interactions between the carbon dots and the analytes [170]. Colorimetric and visual detection systems based on QDs are simple and rapid analytical methods used for on-site and real-time detection of drugs, biomolecules, and chemical substances. Quantum dots possess unique optical properties, such as strong and tunable fluorescence and high sensitivity to their chemical environment.

In these systems, the interaction between quantum dots and the target analyte causes a visible color change or a change in fluorescence color/intensity, which can often be observed with the naked eye or under UV light without using complex instruments. This change may occur due to aggregation, surface reactions, or energy/charge transfer mechanisms. Because of their simplicity, low cost, fast response, and ease of operation, colorimetric and visual detection systems of quantum dots are widely used in rapid screening, quality control, and point-of-care testing.

Aggregation-Induced Quenching (AIQ): In some systems, carbon dots aggregate in the presence of specific analytes, leading to a decrease in fluorescence or a visible color change. This phenomenon is used for the detection of ions, drugs, and biomolecules [171]. Catia Correia et al. synthesized Eu (III)-doped CDs from citric acid and urea via the hydrothermal method, while they used Eu (NO_3_)_3_ as a europium source [172].

**Figure 13:**
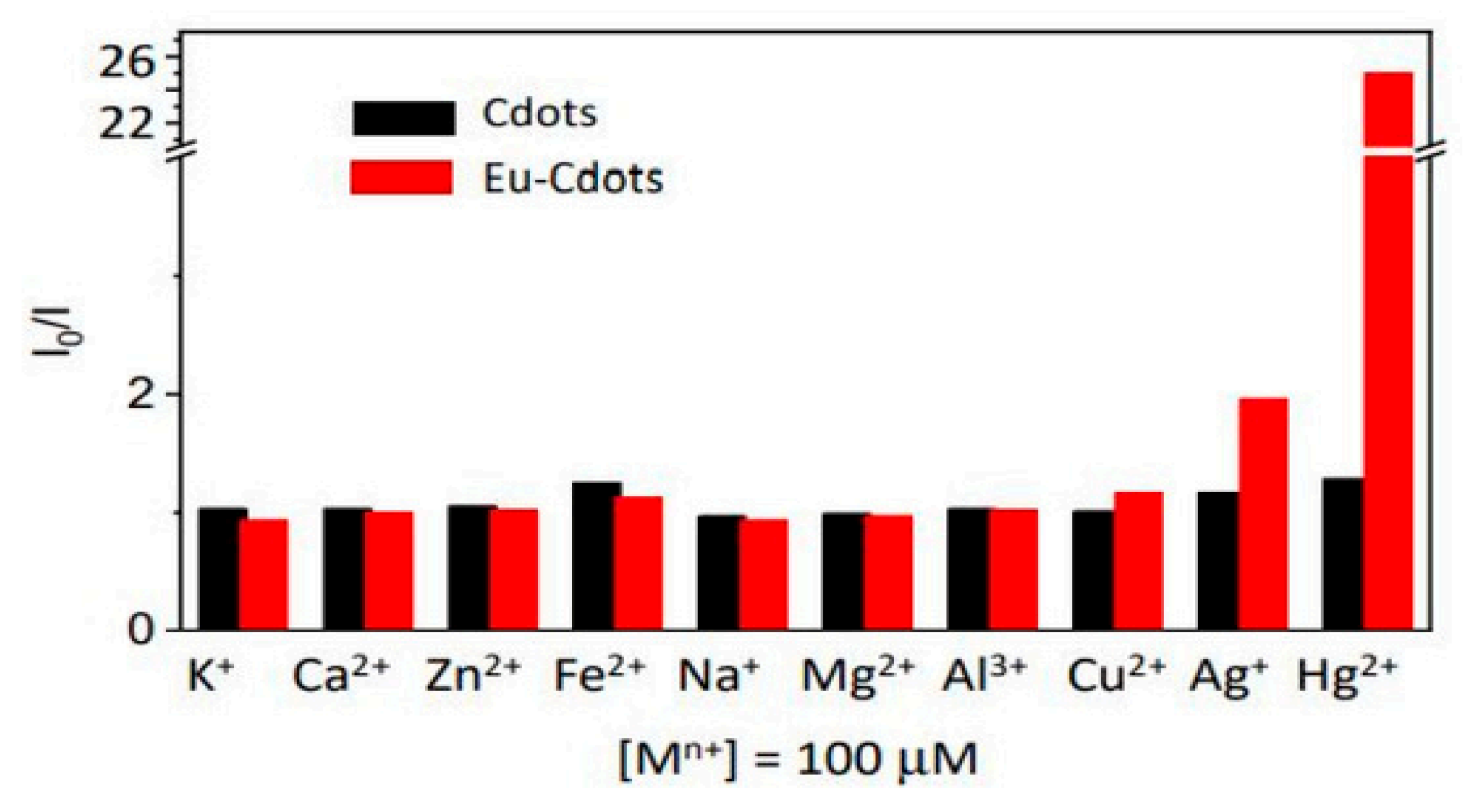
Quenching ratio of various ions with Eu^3+^. Quenching by a factor of 2 is detected for Ag^+^, whereas quenching by a factor of 25 was found for Hg^2+^. Reproduced from [172] under CC BY 4.0 license.

Due to the difference in CD structure and the presence of active sites for cation binding, the detection performance of europium-doped CDs is higher than that of undoped CDs. The quenching impact of Eu^3+^-doped CDs for various metal ions is displayed in Figure 13.

### 7.4. Applications in Pharmaceutical Analysis

Carbon dots can be used to selectively quantify small-molecule drugs in complex biological samples. Their high surface area allows for effective drug adsorption, enabling sensitive detection even at low concentrations [23,173]. Quantum dot-based fluorescent sensing is a powerful, sensitive, and simple technique for the quantification of small-molecule drugs in pharmaceutical formulations and biological samples. For example, CQDs show strong blue fluorescence. When paracetamol is added, the fluorescence is quenched due to the inner filter effect. The decrease in fluorescence intensity is proportional to paracetamol concentration, allowing its quantitative determination in tablets and plasma samples. Other drugs that are commonly quantified are as follows: Aspirin, Ciprofloxacin, Doxorubicin, Tetracycline, Ibuprofen, vitamin B complex, anticancer, and anti-inflammatory drugs [35].

By computing the median and geometric mean LOD value, the biosensors’ performance was assessed using popular fluorescence and colorimetric techniques. For example, the biomarkers utilized in CDs for drug detection include HIV-1 p24 antigen, CEA, PSA, CYFRA 19-1, ATP, NoV-L, 4,4-dibrominated tetracycline, AFP, PCT, VEGF, NMP22, biphenyl, anthrax protective antigen, and fenitrothion. Since fluorescence offers great sensitivity, excellent selectivity, a high signal-to-noise ratio, and quick reaction, it is a popular approach for detecting miRNAs. Although bare carbon dots’ weak ECL emissions and broad excitation potentials restrict their usage in ECL assays, they can be doped with heteroatoms or combined with co-reactants like DNA, hydrogen peroxide, and peroxodisulfate [174].

CDs have gained much popularity in antigen–antibody immunosensors due to their several benefits, including minimal toxicity, high biocompatibility, highly sensitive detection, and strong electrochemical responsiveness [175]. CDs were used in a variety of analytical techniques, including fluorescence, electrochemical approaches, and electrochemiluminescence. It was shown that most non-catalytic protein biomarkers are based on fluorescence techniques [63]. The conductivity of immune complexes formed by CDs enables the biosensor’s electrochemical fluorescence-based functioning mechanism. The analytical performance of articles based on antigen–antibody interactions was reported [63]. Their LOD, linear or dynamic range, and RSD values, including their average and range, are summarized in Table 3. Sulfur-doped agar-derived carbon dots (S-agCDs) are used in surface-enhanced Raman scattering (SERS) techniques to detect norovirus-like particles (Table 4) [176].

The human body uses enzymes, which are biological molecules, to speed up biological reactions and increase their efficiency. Because of their catalytic activity, enzymatic biosensors are more sensitive and may identify analytes with a lower detection limit [180]. Due to the specificity of the enzymes, a variety of enzymatic biosensors employ CDs to detect analytes; nevertheless, enzymes attached to CDs also increase the biosensors’ sensitivity and adaptability.

## 8. CD-Based POC and Miniaturized Biosensors

At present, many diseases are monitored in hospitals and laboratories using traditional diagnostic analysis, which is seen as intrusive, tedious, and costly [184,185]. POC technology based on biomarker testing and monitoring the disease or contaminants is a viable strategy for lowering costs, saving time, and simplifying analysis; enabling go-home patient testing in medical facilities; and enabling the provision of home healthcare services [185,186,187]. In spite of that, many of the clinical methodologies and techniques used today, based on biomarker analysis, have certain limits in terms of cost, size, and integration into portable POC medical devices, even when they are quite exact [186,187]. In this regard, electrochemical and optical detection systems can provide strong downsizing potential, low cost, high sensitivity, and simple integration in small analytical equipment [185,188].

### 8.1. Microfluidic and Lab-on-Chip Platforms

Microfluidic chips, sometimes called Micro Total Analysis System (μTAS), provide an accurate control of tiny fluid volumes (10^−6^–10^−15^ mL) in tens of microliters [189,190]. The concurrent identification of several parallel samples, high capacity, high sensitivity, quick analysis durations, and the use of minimal amounts of samples and reagents are only a few of the many benefits of microfluidic systems [189,190,191]. Numerous fields, including the studies on genomic and proteomic disciplines, medical diagnostics, biohazard identification, analytical chemistry, and environmental monitoring, have made extensive use of microfluidics [190,191]. It has been an emerging field involving interdisciplinary research areas (such as micromechanics, nanotechnology, microelectronics, and bioengineering) [189,190].

Hu et al. created a new microfluidic paper analytical device (μPAD) that effectively combined automatic serum extraction with dependable dual-mode iron health tests: colorimetric ELISA for ferritin and fluorescence analysis for Fe^3+^. In situ CDs and AuNPs sequential patterning techniques provide all these functions. A patterned through-hole polydimethylsiloxane (PDMS) mask was put on paper after a hydrothermal reaction was used to immobilize CDs. On exposed areas, no fluorescence CDs (nF-CDs) were produced, while on covered areas, fluorescent CDs (F-CDs) were produced concurrently. On the F-CD-modified areas, where Fe^3+^ ions can selectively quench the fluorescence of F-CDs, sensitive serum iron detection was achieved. Electroless plating was used on areas modified by nF-CDs to immobilize AuNPs. On the one hand, the resulting AuNPs on the nF-CDs layer caused blood cells to coagulate, resulting in the longest wicking distance for serum separation; on the other hand, they made it easier to detect serum ferritin using the colorimetric enzyme-linked immunosorbent test (ELISA). The μPAD can reliably quantify serum ferritin and iron in whole blood by combining the two values. Additionally, because CDs and AuNPs modified μPAD are lightweight, disposable, inexpensive, and easy to handle, it is a potential prototype for whole blood POC analysis [192]. Wang et al. also fabricated Eu-doped CDs-MOF-based sensors for spore biomarker detection, which also has a prominent feature of the above POC discussed [193].

### 8.2. Paper-Based Analytical Devices

Recently, paper-based gadgets have been at the forefront of analytical division [194]. Li et al. creatively suggested a fluorescent paper-based sensor (FPS) built on a hybrid polydimethylsiloxane (PDMS)/paper platform. Folic acid (FA) was used as the target analyte in an investigation of FPS performance as a proof of concept. Under ideal circumstances, FPS allowed for a quick fluorescence quenching effect to FA through the inner filter effect in a broad range of 1–300 μmol L^−1^ with a 0.28 μmol L^−1^ detection limit. The successful identification of FA in urine and orange juice samples further confirmed the viability of FPS. The covalent alteration of CDs on paper gave the FPS good assay stability and repeatability. Compared to the traditional method, which used the same CDs directly to identify FA in a solution-based system, FPS produced a more sensitive assay of FA. The FPS revealed a new approach to generating sensitive and dependable assays using paper-based instruments. Its practical application in biosensing and clinical diagnostics makes it crucial [195]. FA is used as a precursor in the one-pot solvothermal process used to create the blue-emission CDs (FA-CDs). When irradiated at 360 nm with a QY of 31.2%, the FA-CDs produced strong emission at 445 nm. FA-CDs can be used as fluorescent probes due to their sensitive quenching response to Hg^2+^ with varying concentrations and a detection limit of 1.29 nM. Interestingly, it can be reused about three times, which helps save resources and protect the environment [195]. Rossini and researchers investigated the use of a cotton-paper-syringe filtration system as a quick, easy, and inexpensive pretreatment for saliva samples, enabling the analysis of saliva samples utilizing multilayer paper devices. The suggested process uses certain oxidase enzymes to catalyze the oxidation of glucose and lactate, resulting in the production of hydrogen peroxide. The detection relies on the dampening of CDs’ fluorescence when hydrogen peroxidase is present. With a limit of detection (LOD) of 2.60 × 10^−6^ and 8.14 × 10^−7^ mol L^−1^ for glucose and lactate, respectively. The analyte concentrations demonstrated strong linear correlations with the fluorescence quenching [196]. Such how paper based fluorescent analytical devices have a great advantage of sensitivity and reusability to prompt the detection time with less concentration in real-time applications.

### 8.3. Smartphone-Based Portable Sensing Systems

**Figure 14:**
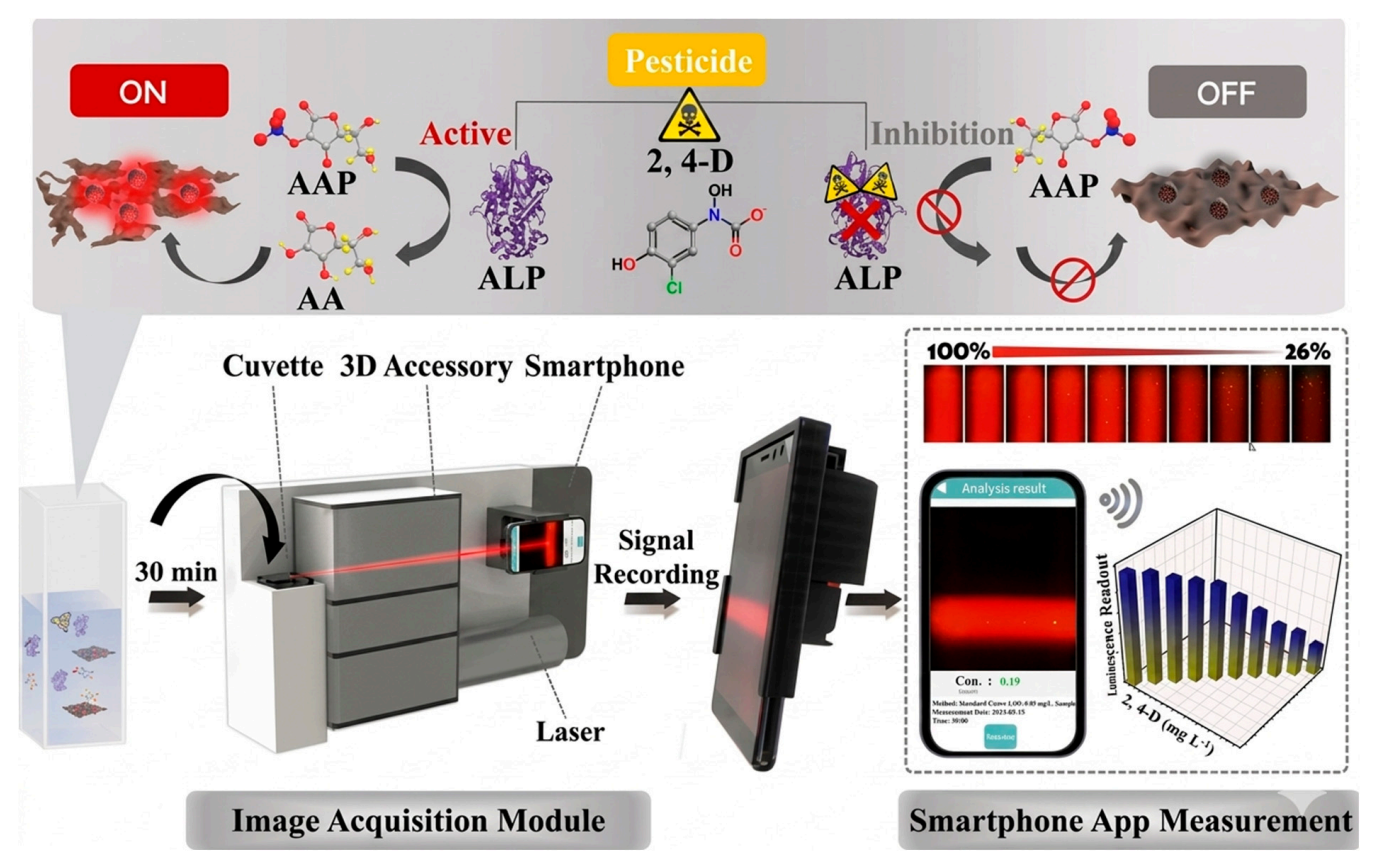
The smartphone-based fluorescent biosensor. It detects the 2,4-D pesticide with the help of an ALP (alkaline phosphatase) inhibitor. In the absence of 2,4-D, the AAP converts into AA with the help of ALP active catalyzes. It is known as the “ON” state and generates strong red fluorescence from sanoprobe. On the contrary, the presence of 2,4-D inhibits the ALP activity known as the “OFF” state and decreases the fluorescence intensity. As shown, the sample will be kept with the 3D-printed smartphone accessories after 30 min of incubation. A lab designed a special mobile software that can show the signal variations with concentration. A specialized mobile application that displays concentration-dependent signal variation and calibration data for 2,4-D detection is used to quantitatively analyze the emitted fluorescence signal, which is captured by a smartphone camera. Reproduced from [200]. Copyright ACS 2020.

Smartphones play a vital role in both daily life and the workplace. Due to their capabilities in communication, photography, analysis, and other areas, mobile phones are an excellent example for portable and smart detection of pharmaceuticals and other contaminants as a low-cost and easy-to-carry system on-site. Moreover, smartphones can easily integrate with sensors like test strips, sensing chips, and other means [197,198]. The smartphones can record the fluorescence color and translate it into red–green–blue (RGB) values for POCT and quantitative analysis of analytes. As a result, cellphones have enormous potential for useful applications in the field of detection. Li et al. fabricated a ratiometric fluorescence sensor. The sensor is based on the mixing of CDs with blue and yellow fluorescence (B-CDs and Y-CDs) to detect Cu^2+^. The smart and intelligent device is made of an optical detector and a smartphone, which is designed for visual POC detection of Cu^2+^ in water and food samples. This approach may create a new tool for food safety and pollution monitoring to detect heavy metals in the industry [62]. Very recently, hybrid CDs made by surface modification of carbazole (Car@CD) and anthracene (Ant@CD) have been used to detect benfluralin, aclonifen, and pendimethalin herbicides fluorometrically in food samples. GC-MS analyses and spike/recovery tests were used to confirm the new procedures. It was observed that the LOD was 6.00–9.40 nM. At the end, both the CDs were used as paper-based testing kits by following the RGB analysis by integrating with a smartphone. The great selectivity of the Car@CD and Ant@CD towards herbicides disclosed accurate detection and fluorometric response. The herbicide recognition was enhanced with the functionalization of CDs with carbazole and anthracene, which can add the π–π interactions and hydrogen bonding. The current strategy provides high sensitivity and real-sample validation. This portable smartphone-assisted strategy gives a paper kit for practical food safety monitoring, given that studies on pendimethalin are still rare and fluorescence-based detection of benfluralin and aclonifen is still nearly nonexistent [199]. Another novel application of smartphone-assisted real-time detection of 2,4-dichlorophenoxyacetic acid (2,4-D) was done by Su et al. using CD/CoOOH (cobalt oxy hydroxide) nanocomposite. The LOD in this study is 100 μg/L. The researchers used a self-made software, which is very interesting and an inspiration for such studies. See the detailed graphical view in Figure 14 [200]. Zhu et al. made red CDs integrated with a smartphone sensor system for the detection of pyrethroids in real time. The LOD of smartphone-based sensor revealed 6.66 μg/L in the concentration ranges of 1–120 μg/L, which is incredible [201]. In this section, we have discussed smartphone-based POC detection for various chemicals, and there is a lot to explore POCs to detect the advanced pharmaceuticals in water, air, soil, and food.

### 8.4. AI-Assisted Chemical Analysis and Validation

**Figure 15:**
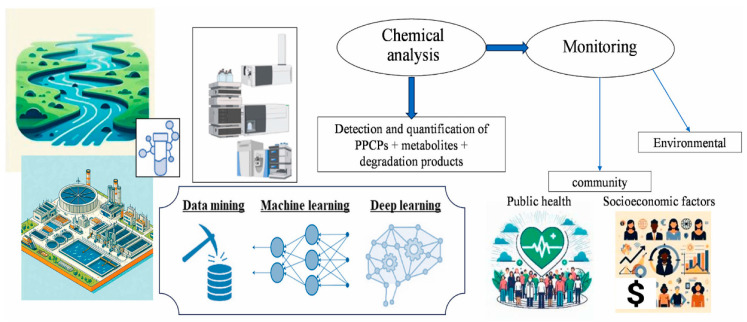
AI-based (data mining, machine learning, and deep learning) strategy for chemical analysis, followed by environmental and community monitoring. Reproduced from [204] under CC BY 4.0 license.

AI-driven approaches based on machine learning, data mining, and deep learning methods integrated with analytical chemistry help automate the identification, categorization, and quantification of complicated chemical compounds easily in a short time with the highest accuracy. In the pharmaceutical and health care industry context, the hidden large data-driven AI models greatly support method validation, quality control, and thereby quality assurance [202,203,204]. AI made a paradigm shift in pharmaceutical quality control and chemical analysis, thereby monitoring the environment throughout the day without losing a single data point, and made a great transformation in the accurate prediction and analysis of complex chemical compounds or trace elements in food, air, soil, and water [204,205]. AI-based chemical analysis has turned out to be very influential in environmental research. It helps to identify various pharmaceutical and personal care products (PPCPs), metabolites, and degradation products, especially in complex matrices like surface water and wastewater or agricultural soils [204,205,206]. Real-time risk evaluation at the community level, automated feature extraction, and predictive modeling of pollutant characteristics could be possible by the combination of deep learning and analytical platforms, as shown in Figure 15 [204]. Regardless of these developments, many of the current applications have stopped at the proof-of-concept stage. Because of the regulatory constraints, limited generalizability, and lack of standardized validation frameworks [204,205,206].

AI-assisted prediction of chromatographic retention periods and collision cross-sections (CCSs) in suspect and non-target screens using high-resolution mass spectrometry (HRMS) improves detection confidence, shortens analytical times, and lowers costs in the chemical analysis of PPCPs. AI also helps interpret the results of spectroscopic analysis. Nevertheless, due to its reduced sensitivity compared to HRMS or liquid chromatography in tandem, this method is still not applicable in all matrices. In order to interpret PPCP monitoring, unsupervised AI techniques have recently shown that they can survey socioeconomic and health aspects in regional or national communities. The lack of long-term monitoring data sources in the literature is an issue, though, and more comparative AI research is needed for both chemical analysis and monitoring. Finally, with AI support, it is expected that data processing for advanced pharmaceuticals available in the environment, wastewater-based epidemiology, and community health surveillance will use AI techniques, and that CCS prediction will be used more frequently to increase detection confidence [204,205,206]. The chances of errors occurring would be rectified by the complete automation of the laboratories and pharmaceutical industries. To achieve the goal of 100% accuracy and great confidence in the results, the chemist does not have to wait for longer times due to the rapid implementation of AI in all sectors.

### 8.5. Clinical and Field-Deployable Pharmaceutical Testing

The CDs could be a good nanoprobe in clinical and field-deployable pharmaceutical testing due to their remarkable properties discussed in the earlier section. Antibiotics like tetracycline, ciprofloxacin, amoxicillin, and chloramphenicol in biological fluids and pharmaceutical formulations have been detected using CD-based fluorescence quenching or ratiometric mechanisms. This facilitates fast therapeutic monitoring and residue analysis [196,198]. CDs have also been efficiently adopted in anticancer drug analysis to identify doxorubicin, methotrexate, and cisplatin. In complex matrices like serum and urine, selective detection is made possible by π–π stacking interactions and electron transfer processes [196,206]. Using CD-based paper sensors and microfluidic platforms, vitamin analysis has been demonstrated, importantly for vitamin B9, vitamin C, and vitamin B2, giving prompt visual or smartphone-assisted readouts appropriate for POC testing [60,191,193,203,207]. In addition, CDs incorporated into lab-on-a-chip systems and paper-based analytical devices have shown a strong potential for detecting subpar and counterfeit drugs, where fluorescence intensity variations allow for quick screening of APIs outside of centralized laboratories [195,201]. These devices are importantly appealing for clinical diagnostics and field inspections in resource-constrained environments since CD integration with microfluidics and smartphone-based imaging systems further improves mobility, quantitative capabilities, and real-time data collection [32].

## 9. CD-Based Sensor Selectivity and Interference in Complex Matrices

CD-based biosensors or chemosensors show very high sensitivity and fast response, yet their selectivity in wastewater, blood, serum, urine, and food extracts remains a major challenge [64,65,188]. Interference occurs from concurrent species like salts, proteins, and metal ions arising from nonspecific adsorption, quenching of fluorescence, autofluorescence, and inner filter effects by matrix-triggered optical effects [11,49,208]. In other words, the CDs’ surface active sites can be passivated or blocked by the high concentration ions, biomacromolecules, which can reduce the target binding sites. These effects produce negative and false positive results [46,47]. Moreover, the PET or FRET signals may be changed by the redox-active interferences in the sample matrix [209,210].

In order to overcome these challenges, PEGylation and zwitterionic coating-based surface passivation of CDs are highly useful. The surface passivation can inhibit the biofouling, biorecognition-triggered selectivity using enzymes, antibodies, or aptamers, and ratiometric sensing to self-correct matrix effects [211,212,213,214]. Incorporating molecular imprinting, dual-signal outputs, or on-chip sample pretreatment (filtration, dilution, and magnetic separation) further enhances discrimination in real samples [33,84,215]. Nevertheless, achieving reproducible selectivity across clinical and environmental matrices demands systematic interference studies, standardized protocols, and validation with certified reference materials, as performance in buffers frequently overestimates real-world analytical reliability [56,185,190,215].

## 10. Limitations

CDs are widely used in pharmaceutical applications. However, despite their advantages (low toxicity, easy synthesis, and good fluorescence), they have several limitations [215,216]. Generally, CDs often exhibit a broad size distribution and lack precise control over shape, crystallinity, and surface defects [216]. This leads to inconsistent optical and electronic properties. Many CDs synthesized via simple hydrothermal or pyrolysis methods show moderate to low fluorescence quantum yield. They had an unclear photoluminescence mechanism. They are not very stable in harsh conditions and usually degrade over time due to sensitive functional groups like -COOH, -OH, and -NH_2_. At higher concentrations, CDs tend to aggregate. Hence, difficulty in controlling uniformity in lab-scale synthesis makes the production of high-purity CDs expensive [217,218].

## 11. Future Perspectives and Challenges

Fluorescence-based carbon dot sensors combine versatile mechanisms with excellent optical properties to achieve sensitive, selective detection—especially valuable in pharmaceutical analysis for small molecules, ions, and biologically relevant compounds. Future work should continue to emphasize ratiometric strategies, in situ real-sample validation, and integration with portable devices to bridge lab research and practical deployment.

High-throughput drug discovery and screening (HTS) aim to rapidly evaluate a large number of compounds to identify potential drug candidates. CQDs have emerged as promising tools in this field due to their strong fluorescence, high photostability, good water solubility, low toxicity, and low cost. In HTS, carbon quantum dots are used as fluorescent probes or sensor platforms, where the interaction between CQDs and test compounds produces a change in fluorescence intensity (quenching or enhancement). Using microplate readers and automated systems, hundreds or thousands of samples can be analyzed simultaneously and rapidly. Thus, carbon quantum dot-based screening systems provide a fast, sensitive, and cost-effective approach for high-throughput drug discovery and screening in modern pharmaceutical research.

## 12. Conclusions

CD-based nanobiosensors have emerged as highly versatile and powerful tools for advanced pharmaceutical analysis due to their distinctive optical and electrochemical properties, good biocompatibility, playable surface chemistry, and easy and economic synthesis. This review discussed and highlighted that CDs enable sensitive and selective detection of APIs, degradation products, impurities, and biomarkers through electrochemical, optical, and dual-mode platforms. The feasibility of facile functionalization with enzymes, antibodies, and aptamers remarkably improves the target specificity and analytical performance in complex biological and environmental matrices.

Moreover, the CD-based miniaturized systems, such as microfluidic devices, paper-based sensors, and smartphone-assisted platforms, show strong potential for POC technology and field-deployable applications. In spite of considerable progress, challenges related to large-scale reproducibility, standardization, long-term stability, and regulatory validation remain the main hurdles to clinical and industrial translation. Overall, CD-based chemo and nanobiosensors represent a promising next-generation sensing technology for pharmaceutical quality control, therapeutic drug monitoring, and real-time health diagnostics.

Further progress in this field will require standardized synthetic procedures, antifouling surface engineering, scalable green synthesis methods, and inclusive performance evaluation in real-sample settings in the future. These challenges must be vanquished if CD-based chemo nanobiosensors are to advance from POC studies to genuine tools in daily use.

## Figures and Tables

**Table 2:** Comparison of normal Raman signals and CD-assisted Raman signals.

S. No.	Feature	Normal Raman	CD-Assisted Raman
1	Sensitivity	Low	Medium High
2	Sample adsorption	Poor	Good
3	Signal strength	Weak	Enhanced
4	Cost	Low	Still Low
5	Biocompatibility		High

**Table 3:** The general methods of CD-based antigen—antibody sensors.

S. No.	Analytical Technique	Median LOD, M	Geometric Mean LOD, M	Biomarkers	Ref. No.
1	Fluorescence	8.33 × 10^−13^	8.49 × 10^−13^	HIV-1 p24 antigen, CEA, PSA, ATP, GLY, 4,4-dibrominated biphenyl, tetracycline, CYFRA 19-1, AFP, PCT, VEGF, NoV-L, NMP22, anthrax protective antigen, fenitrothion	[177,178]
2	Electrochemiluminescence	9.28 × 10^−15^	2.10 × 10^−16^	CEA, PSA, SCCA	[103,179,180]
3	Electrochemical	5.50 × 10^−15^	2.97 × 10^−14^	CA125, TNF-a, CEA, Ag-VD2, CYFRA 21-1, NSE	[181,182]
4	Colorimetric	7.65 × 10^−12^	1.48 × 10^−13^	CEA, Diethyl phthalate,	[183]

**Table 4:** Comparison of Raman signals and SERS signals.

S. No.	Feature	Normal Raman	CD-Assisted Raman
1	Main Mechanism	Chemical/Resonance	Electromagnetic + Chemical
2	Enhancement Factor	10–10^6^	10^6^–10^14^
3	Metal Required	No	Yes (Ag, Au, Cu)
4	Hot Spots	No	Yes
5	Sensitivity	Low–Medium	Ultra-high

## Data Availability

Not applicable.

## Abbreviations

PANI	Polyaniline
PEDOT	Poly(3,4 ethylenedioxythiophene)
PSS	Poly(styrene sulfonate)
PET	Polyethylene terephthalate
LOD	Limit of Detection
MOF	Metal–Organic Framework
CDs	Carbon Dots

## References

[1] Schwarzenbach R.P., Escher B.I., Fenner K., Hofstetter T.B., Johnson C.A., von Gunten U., Wehrli B. (2006). The Challenge of Micropollutants in Aquatic Systems. Science.

[2] Rockström J., Steffen W., Noone K., Persson Å., Chapin F.S., Lambin E.F., Lenton T.M., Scheffer M., Folke C., Schellnhuber H.J. (2009). A Safe Operating Space for Humanity. Nature.

[3] Turner A.P.F. (2013). Biosensors: Sense and Sensibility. Chem. Soc. Rev..

[4] Justino C.I.L., Rocha-Santos T.A.P., Duarte A.C. (2017). Review of Analytical Figures of Merit of Sensors and Biosensors in Clinical Applications. TrAC Trends Anal. Chem..

[5] Ronkainen N.J., Halsall H.B., Heineman W.R. (2010). Electrochemical Biosensors. Chem. Soc. Rev..

[6] Dincer C., Bruch R., Kling A., Dittrich P.S., Urban G.A. (2019). Multiplexed Point-of-Care Testing—xPOCT. Adv. Mater..

[7] Naresh V., Lee N. (2021). A Review on Biosensors and Recent Development of Nanostructured Materials-Enabled Biosensors. Sensors.

[8] Chadha U., Bhardwaj P., Agarwal R., Rawat P., Agarwal R., Gupta I., Panjwani M., Singh S., Ahuja C., Selvaraj S.K. (2022). Recent Progress and Growth in Biosensors Technology: A Critical Review. J. Ind. Eng. Chem..

[9] Varadharajan S., Gadre M., Mathur V., Vasanthan K.S. (2025). Sustainable Integration of Nanobiosensors in Biomedical and Civil Engineering: A Comprehensive Review. ACS Omega.

[10] Baig N., Kammakakam I., Falath W. (2021). Nanomaterials: A Review of Synthesis Methods, Properties, Recent Progress, and Challenges. Mater. Adv..

[11] Zulfajri M., Gedda G., Ulla H., Habibati, Gollavelli G., Huang G.G. (2024). A Review on the Chemical and Biological Sensing Applications of Silver/Carbon Dots Nanocomposites with Their Interaction Mechanisms. Adv. Colloid Interface Sci..

[12] Arduini F., Cinti S., Amine A., Moscone D., Palleschi G. (2016). Biosensors Based on Nanomodified Screen-Printed Electrodes. TrAC Trends Anal. Chem..

[13] Pumera M., Sánchez S., Ichinose I., Tang J. (2007). Nanomaterials for Electrochemical Sensing and Biosensing. Sens. Actuators B Chem..

[14] Ligler F.S., White H.S. (2013). Nanomaterials in Analytical Chemistry. Anal. Chem..

[15] Gorle G., Gollavelli G., Nelli G., Ling Y.-C. (2023). Green Synthesis of Blue-Emitting Graphene Oxide Quantum Dots for In Vitro and In Vivo Nano-Imaging. Pharmaceutics.

[16] Zhu S., Meng Q., Wang L., Zhang J., Song Y., Jin H., Zhang K., Sun H., Wang H., Yang B. (2013). Highly Photoluminescent Carbon Dots for Multicolor Patterning, Sensors, and Bioimaging. Angew. Chem. Int. Ed..

[17] Justino C.I.L., Rocha-Santos T.A.P., Duarte A.C., Rocha-Santos T.A.P. (2013). Advances in Point-of-Care Technologies with Biosensors Based on Carbon Nanotubes. TrAC Trends Anal. Chem..

[18] Vigneshvar S., Sudhakumari C.C., Senthilkumaran B., Prakash H. (2016). Recent Advances in Biosensor Technology for Potential Applications—An Overview. Front. Bioeng. Biotechnol..

[19] Machín A., Márquez F. (2025). Next-Generation Chemical Sensors: The Convergence of Nanomaterials, Advanced Characterization, and Real-World Applications. Chemosensors.

[20] Lemke E.A., Schultz C. (2011). Principles for Designing Fluorescent Sensors and Reporters. Nat. Chem. Biol..

[21] Lamkin-Kennard K.A., Popovic M.B. (2019). Sensors: Natural and Synthetic Sensors. Biomechatronics.

[22] Wang B., Cai H., Waterhouse G.I.N., Qu X., Yang B., Lu S. (2022). Carbon Dots in Bioimaging, Biosensing and Therapeutics. Small Sci..

[23] Jiang W., Zhao Y., Zhu X., Liu H., Sun B. (2021). Carbon Dot-Based Biosensors. Adv. NanoBiomed Res..

[24] Wang L., Gu C., Wu L., Tan W., Shang Z., Tian Y., Ma J. (2024). Recent Advances in Carbon Dots for Electrochemical Sensing and Biosensing: A Systematic Review. Microchem. J..

[25] López J.G., Muñoz M., Arias V., García V., Calvo P.C., Ondo-Méndez A.O., Rodríguez-Burbano D.C., Fonthal F. (2025). Electrochemical and Optical Carbon Dots and Glassy Carbon Biosensors: A Review on Their Development and Applications in Early Cancer Detection. Micromachines.

[26] Nath S. (2024). Advancements in Food Quality Monitoring: Integrating Biosensors. Sustain. Food Technol..

[27] Singh R., Gupta R., Bansal D., Bhateria R., Sharma M. (2024). A Review on Recent Trends and Future Developments in Electrochemical Sensing. ACS Omega.

[28] Terzapulo X., Kassenova A., Loskutova A., Bukasov R. (2025). Carbon Dots: Review of Recent Applications and Perspectives in Bio-Sensing and Biomarker Detection. Sens. Bio-Sens. Res..

[29] Wang Y., Hu A. (2014). Carbon Quantum Dots: Synthesis, Properties and Applications. J. Mater. Chem. C.

[30] Sun Y.-P., Zhou B., Lin Y., Wang W., Fernando K.A.S. (2006). Quantum-Sized Carbon Dots for Bright Photoluminescence. J. Am. Chem. Soc..

[31] Xu X., Ray R., Gu Y., Ploehn H.J., Gearheart L., Raker K., Scrivens W.A. (2004). Electrophoretic Analysis and Purification of Fluorescent Carbon Nanotube Fragments. J. Am. Chem. Soc..

[32] Sen S., Bose A. (2025). Carbon Dots: A Review of Innovations, Applications, Challenges, and Future Prospects. Inorg. Chem. Commun..

[33] Mansuriya B.D., Altintas Z. (2021). Carbon Dots: Classification, Properties, Synthesis, Characterization, and Applications in Health Care—An Updated Review (2018–2021). Nanomaterials.

[34] Nazri N.A.A., Azeman N.H., Luo Y., Bakar A.A.A. (2021). Carbon Quantum Dots for Optical Sensor Applications: A Review. Opt. Laser Technol..

[35] Lodha S.R., Merchant J.G., Pillai A.J., Shah S.A., Shah D.R., Patole S.P. (2024). Carbon Dot-Based Fluorescent Sensors for Pharmaceutical Detection: Current Innovations, Challenges, and Future Prospects. Heliyon.

[36] Caroleo F., Magna G., Naitana M.L., Di Zazzo L., Martini R., Pizzoli F., Muduganti M., Lvova L., Mandoj F., Nardis S. (2022). Advances in Optical Sensors for Persistent Organic Pollutant Environmental Monitoring. Sensors.

[37] Rafiq K., Sadia I., Abid M.Z., Waleed M.Z., Rauf A., Hussain E. (2024). Scientific Insights into the Quantum Dots (QDs)-Based Electrochemical Sensors for State-of-the-Art Applications. ACS Biomater. Sci. Eng..

[38] Paul R., Zhai Q., Roy A.K., Dai L. (2022). Charge Transfer of Carbon Nanomaterials for Efficient Metal-Free Electrocatalysis. Interdiscip. Mater..

[39] Zoric M.R., Singh V., Warren S., Plunkett S., Khatmullin R.R., Chaplin B.P., Glusac K.D. (2019). Electron Transfer Kinetics at Graphene Quantum Dot Assembly Electrodes. ACS Appl. Mater. Interfaces.

[40] Sharma A., Tapadia K., Sahin R., Verma D.K., Otero P., Agrawal I. (2024). Carbon Dots in Sensing: Photoelectrochemical, Electrochemiluminescent, Electrochemical, Colorimetric, and Fluorescent Applications. ACS Symp. Ser..

[41] Sato K., Katakami R., Iso Y., Isobe T. (2022). Surface-Modified Carbon Dots with Improved Photoluminescence Quantum Yield for Color Conversion in White-Light-Emitting Diodes. ACS Appl. Nano Mater..

[42] Shabbir H., Csapó E., Wojnicki M. (2023). Carbon Quantum Dots: The Role of Surface Functional Groups and Proposed Mechanisms for Metal Ion Sensing. Inorganics.

[43] Park Y., Yoo J., Lim B., Kwon W., Rhee S.-W. (2016). Improving the Functionality of Carbon Nanodots: Doping and Surface Functionalization. J. Mater. Chem. A.

[44] Wang H., Ai L., Song Z., Nie M., Xiao J., Li G., Lu S. (2023). Surface Modification Functionalized Carbon Dots. Chem. Eur. J..

[45] Rajendran S., UshaVipinachandran V., Badagoppam Haroon K.H., Ashokan I., Bhunia S.K. (2022). A Comprehensive Review on Multi-Colored Emissive Carbon Dots as Fluorescent Probes for the Detection of Pharmaceutical Drugs in Water. Anal. Methods.

[46] Havrdova M., Hola K., Skopalik J., Tomankova K., Petr M., Cepe K., Polakova K., Tucek J., Bourlinos A.B., Zboril R. (2016). Toxicity of Carbon Dots—Effect of Surface Functionalization on the Cell Viability, Reactive Oxygen Species Generation and Cell Cycle. Carbon.

[47] Emam A.N., Loutfy S.A., Mostafa A.A., Awad H., Mohamed M.B. (2017). Cyto-Toxicity, Biocompatibility and Cellular Response of Carbon Dots–Plasmonic Based Nano-Hybrids for Bioimaging. RSC Adv..

[48] Fu Z., Zhang T., Chen C., Wang X., Wang L. (2025). Copper-Based Biomimetic Nanozymes with Multi-Enzyme Activity for Phosphate Detection. Spectrochim. Acta Part A Mol. Biomol. Spectrosc..

[49] Dua S., Kumar P., Pani B., Kaur A., Khanna M., Bhatt G. (2023). Stability of Carbon Quantum Dots: A Critical Review. RSC Adv..

[50] Perikala M., Bhardwaj A. (2019). Highly Stable White-Light-Emitting Carbon Dot Synthesis Using a Non-Coordinating Solvent. ACS Omega.

[51] Amezaga Gonzalez M.F., Ramirez-Reyes A., Mendoza-Duarte M.E., Vega-Rios A., Martinez-Ozuna D., Rodriguez-Gonzalez C.A., Martel-Estrada S.-A., Olivas-Armendariz I. (2025). Stability of Carbon Quantum Dots for Potential Photothermal and Diagnostic Applications. C J. Carbon Res..

[52] Baker S.N., Baker G.A. (2010). Luminescent Carbon Nanodots: Emergent Nanolights. Angew. Chem. Int. Ed..

[53] Lim S.Y., Shen W., Gao Z. (2015). Carbon Quantum Dots and Their Applications. Chem. Soc. Rev..

[54] Qureshi Z.A., Dabash H., Ponnamma D., Abbas M.K.G. (2024). Carbon Dots as Versatile Nanomaterials in Sensing and Imaging: Efficiency and Beyond. Heliyon.

[55] Zhang Q., Wang R., Feng B., Zhong X., Ostrikov K. (2021). Photoluminescence Mechanism of Carbon Dots: Triggering High-Color-Purity Red Fluorescence Emission through Edge Amino Protonation. Nat. Commun..

[56] Huang Z., Ren L. (2025). Large Scale Synthesis of Carbon Dots and Their Applications: A Review. Molecules.

[57] Xu W., Zeng F., Han Q., Peng Z. (2024). Recent advancements of solid-state emissive carbon dots: A review. Coord. Chem. Rev..

[58] Wang Q., He X., Lv Y., Lyu Y., Liu J., Wang L., Yang S., Yan A., Wang S., Guo P. (2025). A Review of Carbon Dots in Synthesis, Property and Application. Mater. Today Commun..

[59] Ding H., Xiao T., Ren F., Qiu Y., Shen Z., Chen X. (2024). Carbon-Based Nanodots for Biomedical Applications. BMEMat.

[60] Varadharajan S., Vasanthan K.S., Mathur V., Hariperumal N., Mazumder N. (2024). Green Synthesis and Multifaceted Applications: Challenges and Innovations in Carbon Dot Nanocomposites. Discov. Nano.

[61] Etefa H.F., Tessema A.A., Dejene F.B. (2024). Carbon Dots for Future Prospects: Synthesis, Characterizations and Recent Applications: A Review (2019–2023). C J. Carbon Res..

[62] Li T., Wu T., Lu M., Li N., Ma Y., Song L., Huang X., Zhao J., Wang T. (2024). An Intelligent Device with Double Fluorescent Carbon Dots Based on Smartphone for Visual and Point-of-Care Testing of Copper(II) in Water and Food Samples. Food Chem. X.

[63] Barrientos K., Arango J.P., Moncada M.S., Placido J., Patiño J., Macías S.L., Maldonado C., Torijano S., Bustamante S., Londoño M.E. (2023). Carbon dot-based biosensors for the detection of communicable and non-communicable diseases. Talanta.

[64] Mir T.U.G., Wani A.K., Akhtar N., Katoch V., Shukla S., Kadam U.S., Hong J.C. (2023). Advancing Biological Investigations Using Portable Sensors for Detection of Sensitive Samples. Heliyon.

[65] Redondo-Fernandez G., Cigales Canga J., Soldado A., Ruiz Encinar J., Costa-Fernandez J.M. (2023). Functionalized Heteroatom-Doped Carbon Dots for Biomedical Applications: A Review. Anal. Chim. Acta.

[66] Khasim S., Al-Ghamdi S.A., Darwish A.A.A., Hamdalla T.A., Pasha A. (2023). Biosynthesis of Carbon Quantum Dot Nanocomposite as an Advanced Material for Simultaneous Electrochemical Sensing of D-Glucose and Paracetamol. Appl. Phys. A.

[67] Murugesan A., Li H., Shoaib M. (2025). Recent Advances in Functionalized Carbon Quantum Dots Integrated with Metal–Organic Frameworks: Emerging Platforms for Sensing and Food Safety Applications. Foods.

[68] Miao H., Wang P., Cong Y., Dong W., Li L. (2023). Preparation of Ciprofloxacin-Based Carbon Dots with High Antibacterial Activity. Int. J. Mol. Sci..

[69] Arora G., Sabran N.S., Ng C.Y., Low F.W., Jun H.K. (2024). Applications of Carbon Quantum Dots in Electrochemical Energy Storage Devices. Heliyon.

[70] Li H., Kang Z., Liu Y., Lee S.-T. (2012). Carbon Nanodots: Synthesis, Properties and Applications. J. Mater. Chem..

[71] Hatimuria M., Phukan P., Bag S., Ghosh J., Gavvala K., Pabbathi A., Das J. (2023). Green Carbon Dots: Applications in Development of Electrochemical Sensors, Assessment of Toxicity as Well as Anticancer Properties. Catalysts.

[72] Malode S.J., Alshehri M.A., Shetti N.P. (2024). Nanomaterial-Based Electrochemical Sensors for the Detection of Pharmaceutical Drugs. Chemosensors.

[73] Kar D.K., Praveenkumar V., Si S., Panigrahi H., Mishra S. (2024). Carbon Dots and Their Polymeric Nanocomposites: Insight into Their Synthesis, Photoluminescence Mechanisms, and Recent Trends in Sensing Applications. ACS Omega.

[74] Aihaiti A., Li Z., Qin Y., Meng F., Li X., Huangfu Z., Chen K., Zhang M. (2022). Construction of Electrochemical Sensors for Antibiotic Detection Based on Carbon Nanocomposites. Nanomaterials.

[75] Leau S.-A., Lete C., Lupu S. (2023). Nanocomposite Materials Based on Metal Nanoparticles for the Electrochemical Sensing of Neurotransmitters. Chemosensors.

[76] Khalilzadeh A., Soleymanpour A., Zarei K. (2025). Gold Nanoparticles @ Nitrogen-Doped Carbon Dots Modified Pencil Graphite Electrode as an Extremely Sensitive Sensor for Trace Analysis of Ciprofloxacin. J. Nanopart. Res..

[77] Tiwari J.N., Vij V., Kemp K.C., Kim K.S. (2016). Engineered Carbon-Nanomaterial-Based Electrochemical Sensors for Biomolecules. ACS Nano.

[78] Zhang L., He L., Wang Q., Tang Q., Liu F. (2020). Theoretical and Experimental Studies of a Novel Electrochemical Sensor Based on Molecularly Imprinted Polymer and GQDs-PtNPs Nanocomposite. Microchem. J..

[79] Lupu S. (2025). Polymeric Composite-Based Electrochemical Sensing Devices Applied in the Analysis of Monoamine Neurotransmitters. Biosensors.

[80] Bounegru A.V., Iacob A.D., Iticescu C., Georgescu P.L. (2025). Electrochemical Sensors and Biosensors for the Detection of Pharmaceutical Contaminants in Natural Waters—A Comprehensive Review. Chemosensors.

[81] Palsaniya S., Pal T., Mukherji S. (2023). Highly Sensitive Detection of Amoxicillin by Polyaniline-AgBr Amperometry Sensor: Fabrication and Application in Tap Water and Lake Water. Chem. Eng. J..

[82] Abdel-Aal F.A.M., Kamel R.M., Abdeltawab A.A., Mohamed F.A., Mohamed A.-M.I. (2023). Polypyrrole/Carbon Dot Nanocomposite as an Electrochemical Biosensor for Liquid Biopsy Analysis of Tryptophan in the Human Serum of Normal and Breast Cancer Women. Anal. Bioanal. Chem..

[83] Pal A., Sk M.P., Chattopadhyay A. (2016). Conducting Carbon Dot–Polypyrrole Nanocomposite for Sensitive Detection of Picric Acid. ACS Appl. Mater. Interfaces.

[84] Jing H.H., Adnan M., Patel M., Sasidharan S. (2025). Reproducibility Roadblocks and Standardization in Carbon Dot Synthesis: A Critical Review of Current Practices, Challenges, and Future Directions. Microchim Acta.

[85] Li M., Wang W., Chen Z., Song Z., Luo X. (2018). Electrochemical Determination of Paracetamol Based on Au@graphene Core-Shell Nanoparticles Doped Conducting Polymer PEDOT Nanocomposite. Sens. Actuators B Chem..

[86] Santos A.M., Feitosa M.H.A., Wong A., Fatibello-Filho O., Sotomayor M.D.P.T., Moraes F.C. (2024). Functionalized Graphene, Quantum Dots, and PEDOT:PSS Based Screen-Printed Electrode for the Endocrine Disruptor Bisphenol A Determination. Sens. Actuators B Chem..

[87] Cayuela A., Soriano M.L., Carrillo-Carrión C., Valcárcel M. (2016). Semiconductor and Carbon-Based Fluorescent Nanodots: The Need for Consistency. Chem. Commun..

[88] Xu Q., Su R., Chen Y., Theruvakkattil Sreenivasan S., Li N., Zheng X., Zhu J., Pan H., Li W., Xu C. (2018). Metal Charge Transfer Doped Carbon Dots with Reversibly Switchable, Ultra-High Quantum Yield Photoluminescence. ACS Appl. Nano Mater..

[89] Rizk M., Ramzy E., Toubar S., Mahmoud A.M., El Hamd M.A., Alshehri S., Helmy M.I. (2024). Bioinspired Carbon Dots-Based Fluorescent Sensor for the Selective Determination of a Potent Anti-Inflammatory Drug in the Presence of Its Photodegradation Products. ACS Omega.

[90] Zhang D.-H., Yang L., Li N., Su K., Liu L., Li C.-Y. (2024). Detection of Ciprofloxacin and pH by Carbon Dots and Rapid, Visual Sensing Analysis. Food Chem..

[91] Srivastava A.K., Upadhyay S.S., Rawool C.R., Punde N.S., Rajpurohit A.S. (2019). Voltammetric techniques for the analysis of drugs using nanomaterials based chemically modified electrodes. Curr. Anal. Chem..

[92] Al-mashriqi H.S., Zhang Y., Chen J., Qaed E., Li X., Yang Y., Qiu H. (2025). Fabrication of Chiral Fluorescence Carbon Dots-Based Nanosensor for Selective Sensing of L-Cysteine, Antibiotic Drug in Biological Fluid, and Bioimaging Application. Mater. Today Chem..

[93] Baj-Rossi C., Rezzonico Jost T., Cavallini A., Grassi F., De Micheli G., Carrara S. (2014). Continuous Monitoring of Naproxen by a Cytochrome P450-Based Electrochemical Sensor. Biosens. Bioelectron..

[94] Canevari T.C., Cincotto F.H., Nakamura M., Machado S.A.S., Toma H.E. (2016). Efficient Electrochemical Biosensors for Ethynylestradiol Based on the Laccase Enzyme Supported on Single Walled Carbon Nanotubes Decorated with Nanocrystalline Carbon Quantum Dots. Anal. Methods.

[95] Chellachamy Anbalagan A., Korram J., Doble M., Sawant S.N. (2024). Bio-Functionalized Carbon Dots for Signaling Immuno-Reaction of Carcinoembryonic Antigen in an Electrochemical Biosensor for Cancer Biomarker Detection. Discov. Nano.

[96] Evtugyn G., Porfireva A., Shamagsumova R., Hianik T. (2020). Advances in Electrochemical Aptasensors Based on Carbon Nanomaterials. Chemosensors.

[97] Campuzano S., Yáñez-Sedeño P., Pingarrón J.M. (2019). Carbon Dots and Graphene Quantum Dots in Electrochemical Biosensing. Nanomaterials.

[98] Li F., Xiong S., Tan Y., Luo M., Wu Z. (2025). A Simple Self-Assembly Aptasensor for Ultrasensitive Detection of Kanamycin Based on Carbon Dots and Ti3C2 MXene Nanocomposite. RSC Adv..

[99] Mat Zaid M.H., Abdullah J., Rozi N., Mohamad Rozlan A.A., Abu Hanifah S. (2020). A Sensitive Impedimetric Aptasensor Based on Carbon Nanodots Modified Electrode for Detection of 17ß-Estradiol. Nanomaterials.

[100] Majumdar S., Thakur D., Chowdhury D. (2020). DNA Carbon-Nanodots Based Electrochemical Biosensor for Detection of Mutagenic Nitrosamines. ACS Appl. Bio Mater..

[101] Mahmoudi-Moghaddam H., Tajik S., Beitollahi H. (2019). A New Electrochemical DNA Biosensor Based on Modified Carbon Paste Electrode Using Graphene Quantum Dots and Ionic Liquid for Determination of Topotecan. Microchem. J..

[102] Kim Y.S., Jung H.S., Matsuura T., Lee H.Y., Kawai T., Gu M.B. (2007). Electrochemical Detection of 17β-Estradiol Using DNA Aptamer Immobilized Gold Electrode Chip. Biosens. Bioelectron..

[103] Li R., Wang C., Hu Y., Zheng O., Guo L., Lin Z., Qiu B., Chen G. (2014). Electrochemiluminescence Biosensor for Folate Receptor Based on Terminal Protection of Small-Molecule-Linked DNA. Biosens. Bioelectron..

[104] Ensafi A.A., Nasr-Esfahani P., Rezaei B. (2018). Metronidazole Determination with an Extremely Sensitive and Selective Electrochemical Sensor Based on Graphene Nanoplatelets and Molecularly Imprinted Polymers on Graphene Quantum Dots. Sens. Actuators B Chem..

[105] Hassanvand Z., Jalali F., Nazari M., Parnianchi F., Santoro C. (2021). Carbon Nanodots in Electrochemical Sensors and Biosensors: A Review. ChemElectroChem.

[106] Kim J.Y., Kim M.Y., Song Y., Oh M.J., Bong J.-H., Park M. (2026). Recent Strategies in Nanomaterials-Based Signal Amplification of Electrochemical Biosensors. BioChip J..

[107] Gamboa J., el Attar R., Thuau D., Estrany F., Abbas M., Torras J. (2024). Carbon Quantum Dots Composite for Enhanced Selective Detection of Dopamine with Organic Electrochemical Transistors. Microchim. Acta.

[108] Smajdor J., Paczosa-Bator B., Piech R. (2023). Electrochemical Sensor Based on the Hierarchical Carbon Nanocomposite for Highly Sensitive Ciprofloxacin Determination. Membranes.

[109] Zhu T., Cao L., Kou X., Liu Y., Dong W.-F., Ge M., Li L. (2022). Nitrogen-Doped Cyan-Emissive Carbon Quantum Dots for Fluorescence Tetracycline Detection and Lysosome Imaging. RSC Adv..

[110] Cheng Y., Wang X., Mei Y., Wang D., Ji C. (2021). ZnCDs/ZnO@ZIF-8 Zeolite Composites for the Photocatalytic Degradation of Tetracycline. Catalysts.

[111] Nugroho D., Wannakan K., Nanan S., Benchawattananon R. (2024). The Synthesis of Carbon Dots//Zincoxide (CDs/ZnO-H400) by Using Hydrothermal Methods for Degradation of Ofloxacin Antibiotics and Reactive Red Azo Dye (RR141). Sci. Rep..

[112] Yang L., Huang N., Lu Q., Liu M., Li H., Zhang Y., Yao S. (2016). A Quadruplet Electrochemical Platform for Ultrasensitive and Simultaneous Detection of Ascorbic Acid, Dopamine, Uric Acid and Acetaminophen Based on a Ferrocene Derivative Functional Au NPs/Carbon Dots Nanocomposite and Graphene. Anal. Chim. Acta.

[113] Singh R., Choudhary G., Sharma A., Mohiuddin I., Bhogal S. (2025). Magnetic Layered Double Hydroxides Intercalated with Polydopamine-Modified Carbon Dots for the Detection of Nonsteroidal Anti-Inflammatory Drugs by Spectrofluorometric Analysis. ACS Appl. Nano Mater..

[114] Zhang W., Han D., Wu Z., Yang K., Sun S., Wen J. (2023). Metal-Organic Layers-Catalyzed Amplification of Electrochemiluminescence Signal and Its Application for Immunosensor Construction. Sens. Actuators B Chem..

[115] Brito T.P., Llanos L., Singh D.P. (2026). Carbon Dots and Metal–Organic Frameworks Based Nanohybrids for Improved Biosensing and Biomedical Applications. Nanoscale Adv..

[116] Liang X., Hu Y., Zheng X., Shao Y., Hua Y., Liu J., Zhu Z., Shao Y. (2023). Functionalized Metal–Organic Frameworks Based on Multi-Catalyst Ordered Assembly for Electrochemical Stripping Chemiluminescent Immunoassay. Sens. Diagn..

[117] Di Matteo P., Trani A., Bortolami M., Feroci M., Petrucci R., Curulli A. (2023). Electrochemical Sensing Platform Based on Carbon Dots for the Simultaneous Determination of Theophylline and Caffeine in Tea. Sensors.

[118] Gissawong N., Srijaranai S., Boonchiangma S., Uppachai P., Seehamart K., Jantrasee S., Moore E., Mukdasai S. (2021). An Electrochemical Sensor for Voltammetric Detection of Ciprofloxacin Using a Glassy Carbon Electrode Modified with Activated Carbon, Gold Nanoparticles and Supramolecular Solvent. Microchim. Acta.

[119] Singh T.A., Sharma V., Thakur N., Tejwan N., Sharma A., Das J. (2023). Selective and Sensitive Electrochemical Detection of Doxorubicin via a Novel Magnesium Oxide/Carbon Dot Nanocomposite Based Sensor. Inorg. Chem. Commun..

[120] Thakur N., Sharma V., Singh T.A., Pabbathi A., Das J. (2022). Fabrication of Novel Carbon Dots/Cerium Oxide Nanocomposites for Highly Sensitive Electrochemical Detection of Doxorubicin. Diam. Relat. Mater..

[121] Aslam H.K., Bilal S., Mir S., Tabassum S., Gilani M.A., Yaqub M., Asim M. (2024). A Robust and Simple Non-Enzymatic Electrochemical Sensor Based on Carbon Dots-Metal Oxide Composite for the Detection of Metronidazole Traces in Food Products. Food Chem..

[122] Rateb A., Ghubish Z., Abdel Hakiem A.F., El-Kemary M. (2023). A Multifunctional Sensing of Two Carbon Dots Based on Diaminonaphthalenes for Detection of Ofloxacin Drug. J. Photochem. Photobiol. A Chem..

[123] Feng Y., Li Y., Yu S., Yang Q., Tong Y., Ye B.-C. (2021). Electrochemical Sensor Based on N-Doped Carbon Dots Decorated with Manganese Oxide Nanospheres for Simultaneous Detection of p-Aminophenol and Paracetamol. Analyst.

[124] Silva L.F., Caetano M.M., de Lima R.G. (2023). Simple and Cheap Preparation of Fluorescence Paper Sensor Based in Carbon Dot for Visual Detection of Chloramphenicol. Luminescence.

[125] Vijeata A., Chaudhary G.R., Chaudhary S., Umar A., Akbar S., Baskoutas S. (2023). Label Free Dual-Mode Sensing Platform for Trace Level Monitoring of Ciprofloxacin Using Bio-Derived Carbon Dots and Evaluation of Its Antioxidant and Antimicrobial Potential. Microchim Acta.

[126] Wang J., Lu T., Hu Y., Wang X., Wu Y. (2020). A Label-Free and Carbon Dots Based Fluorescent Aptasensor for the Detection of Kanamycin in Milk. Spectrochim. Acta Part A Mol. Biomol. Spectrosc..

[127] Kundu A., Maity B., Basu S. (2022). Rice Husk-Derived Carbon Quantum Dots-Based Dual-Mode Nanoprobe for Selective and Sensitive Detection of Fe3+ and Fluoroquinolones. ACS Biomater. Sci. Eng..

[128] Fu Y., Huang L., Zhao S., Xing X., Lan M., Song X. (2021). A Carbon Dot-Based Fluorometric Probe for Oxytetracycline Detection Utilizing a Förster Resonance Energy Transfer Mechanism. Spectrochim. Acta Part A Mol. Biomol. Spectrosc..

[129] Le T.H., Lee H.J., Kim J.H., Park S.J. (2020). Highly Selective Fluorescence Sensor Based on Graphene Quantum Dots for Sulfamethoxazole Determination. Materials.

[130] M. A A., Joseph R., Kutti Rani S., Vasimalai N. (2024). A Novel Molecular Imprinted Polymer Grafted on N, S Co-Doped Carbon Quantum Dots-Based Fluorescence Sensor for Chloramphenicol in Food and Clinical Samples. Microchem. J..

[131] Xue J., Li N.-N., Zhang D.-M., Bi C.-F., Xu C.-G., Shi N.-N., Zhang X., Fan Y.-H. (2020). One-Step Synthesis of a Carbon Dot-Based Fluorescent Probe for Colorimetric and Ratiometric Sensing of Tetracycline. Anal. Methods.

[132] Kaurav H., Verma D., Bansal A., Kapoor D.N., Sheth S. (2023). Progress in Drug Delivery and Diagnostic Applications of Carbon Dots: A Systematic Review. Front. Chem..

[133] Laghlimi C., Moutcine A., Chtaini A., Isaad J., Soufi A., Ziat Y., Amhamdi H., Belkhanchi H. (2023). Recent Advances in Electrochemical Sensors and Biosensors for Monitoring Drugs and Metabolites in Pharmaceutical and Biological Samples. ADMET DMPK.

[134] Wei J., Zhang X., Mugo S.M., Zhang Q. (2022). A Portable Sweat Sensor Based on Carbon Quantum Dots for Multiplex Detection of Cardiovascular Health Biomarkers. Anal. Chem..

[135] Xu Z., Song J., Liu B., Lv S., Gao F., Luo X., Wang P. (2021). A Conducting Polymer PEDOT:PSS Hydrogel Based Wearable Sensor for Accurate Uric Acid Detection in Human Sweat. Sens. Actuators B Chem..

[136] Ates H.C., Nguyen P.Q., Gonzalez-Macia L., Morales-Narváez E., Güder F., Collins J.J., Dincer C. (2022). End-to-End Design of Wearable Sensors. Nat. Rev. Mater..

[137] Liu Y., Liu X., Wang X., Jiang H. (2025). AI-Empowered Electrochemical Sensors for Biomedical Applications: Technological Advances and Future Challenges. Biosensors.

[138] Jarosińska E., Zambrowska Z., Witkowska Nery E. (2024). Methods of Protection of Electrochemical Sensors against Biofouling in Cell Culture Applications. ACS Omega.

[139] Das S., Saha B., Tiwari M., Tiwari D.K. (2023). Diagnosis of Cancer Using Carbon Nanomaterial-Based Biosensors. Sens. Diagn..

[140] Azodo A.P., Mezue T.C., Omokaro I. (2025). Smartphone-Based Biosensors: Current Trends, Challenges, and Future Prospects. Eng. Proc..

[141] Nenashev G.V., Istomina M.S., Kryukov R.S., Kondratev V.M., Shcherbakov I.P., Petrov V.N., Moshnikov V.A., Aleshin A.N. (2022). Effect of Carbon Dots Concentration on Electrical and Optical Properties of Their Composites with a Conducting Polymer. Molecules.

[142] Manayil Parambil A., Rajamani P. (2024). Carbon Dots: A Promising Path towards Environmental Sustainability. Environ. Sci. Adv..

[143] Sahu Y., Hashmi A., Patel R., Singh A.K., Susan M.A.B.H., Carabineiro S.A.C. (2022). Potential Development of N-Doped Carbon Dots and Metal-Oxide Carbon Dot Composites for Chemical and Biosensing. Nanomaterials.

[144] Yalshetti S., Thokchom B., Bhavi S.M., Singh S.R., Patil S.R., Harini B.P., Sillanpää M., Manjunatha J.G., Srinath B.S., Yarajarla R.B. (2024). Microwave-Assisted Synthesis, Characterization and In Vitro Biomedical Applications of *Hibiscus rosa-sinensis* Linn.-Mediated Carbon Quantum Dots. Sci. Rep..

[145] Mokti M.H., Tajuddin H.A., Abdullah Z., Hisham S., Mohd Yusof Chan N.N., Idris A., Maher S. (2023). Rapid and Facile Synthesis of High-Color Purity Solid-State Carbon Dots with High Yield Using a Direct Heating Method. Appl. Phys. A.

[146] Overview of IVD Regulation. https://www.fda.gov/medical-devices/ivd-regulatory-assistance/overview-ivd-regulation.

[147] Hu Z., Zhu R., Figueroa-Miranda G., Feng L., Offenhäusser A., Mayer D. (2025). Potential-Pulse-Assisted Co-Immobilization of Multiple Aptamers on Microelectrode Arrays for Multiplexed Neurotransmitter Detection. Biosens. Bioelectron..

[148] Chandran M., Chellasamy G., Veerapandian M., Dhanasekaran B., Govindaraju S., Yun K. (2025). Instant Synthesis of Nitrogen-Doped Ti_3_C_2_ MXene Quantum Dots for Fluorescence and Electrochemical Dual-Mode Detection of Norepinephrine with a Portable Smartphone Assay. J. Mater. Chem. B.

[149] Seo G., Kim B.-S., Lim H., Choi J., Kim M., Lee H., Kim H.-O. (2026). Biomedical applications and future perspectives of carbon dots and their hybrid nanomaterials. Mater. Adv..

[150] Lou X.T., Zhan L., Chen B.B. (2025). Recent Progress of Carbon Dots in Fluorescence Sensing. Inorganics.

[151] Fan Y., Qiao W., Long W., Chen H., Fu H., Zhou C., She Y. (2022). Detection of Tetracycline Antibiotics Using Fluorescent “Turn-Off” Sensor Based on S,N-Doped Carbon Quantum Dots. Spectrochim. Acta Part A Mol. Biomol. Spectrosc..

[152] Ping L., Jun A. (2023). Fluorescence Probes and Their Sensing Applications in Nanomaterials System. Talanta Open.

[153] Sun H., Zhang W., Xie J., Sun M., Hu P., Zhang Z., Zhu J., Zhao Y., Liu L. (2025). IFE and Dynamic Quenching Mediated Fluorescent Sensing of Cr(VI) Based on Nitrogen-Doped Biomass Carbon Dots. Environ. Res..

[154] Silva M.S.A., Camargo A.S.S. (2021). Exploring the Use of Upconversion Nanoparticles in Chemical and Biological Sensors. Nanoscale Adv..

[155] Zhang Y. (2021). Nanomaterial-Based Dual-Emission Ratiometric Fluorescent Sensors for Biosensing and Cell Imaging. Polymers.

[156] Zhai Y., Huang M., Jiang L., Liao H. (2021). Ratiometric Fluorescence Detection of 6-Mercaptopurine Based on the Nanohybrid of Fluorescence Carbon Dots and Gold Nanoclusters. J. Sens. Technol..

[157] Soleja N., Mohd M. (2024). Exploring the Landscape of FRET-Based Molecular Sensors. Biotechnol. Adv..

[158] Yang W.-C., Li S.-Y., Niu S., Liu G. (2024). Advances in FRET-Based Biosensors. Aggregate.

[159] Ren T. (2021). Conformation-Based Stimuli-Response Sensors. Sens. Actuators Rep..

[160] Nayem H. (2024). Prospects and Challenges of Sensor Materials. e-Prime.

[161] Wu L., Huang C., Emery B.P., Sedgwick A.C., Bull S.D., He X.P., Tian H., Yoon J., Sessler J.L., James T.D. (2020). Förster Resonance Energy Transfer (FRET)-Based Small-Molecule Sensors and Imaging Agents. Chem. Soc. Rev..

[162] Fang C., Huang Y., Zhao Y. (2023). Review of FRET biosensing and its application in biomolecular detection. Am. J. Transl. Res..

[163] Ahmed S., Cirone J., Chen A. (2019). Fluorescent Fe3O4 Quantum Dots for H2O2 Detection. ACS Appl. Nano Mater..

[164] Ma Y. (2021). Multi-Carbon Dots and Aptamer Based Ratiometric Fluorescence Probe. J. Nanobiotechnol..

[165] Hao M. (2024). Surface-enhanced Raman spectroscopy. ACS Nano.

[166] Geng P., Wang Y., Peng Z., Hu C., Zhang S., Rao X., Chen G., Mi F., Guan M. (2026). Dual-Driven by Synergistic Enhancement and an Intrinsic Raman Internal Standard: A Multifunctional Nanocomposite Substrate for Ratiometric SERS Detection and Photocatalytic Degradation of Fluoroquinolone Antibiotics. Chem. Eng. J..

[167] Jones R. (2019). Raman Techniques: Fundamentals and Frontiers. Nanoscale Res. Lett..

[168] Cialla-May D., Krafft C., Rösch P., Deckert-Gaudig T., Frosch T., Jahn I.J., Pahlow S., Stiebing C., Meyer-Zedler T., Bocklitz T. (2022). Raman Spectroscopy and Imaging in Bioanalytics. Anal. Chem..

[169] Yang Y. (2025). High SERS Performance of Functionalized Carbon Dots. J. Adv. Res..

[170] Guo S. (2025). Colorimetric Identification Chips for Detecting Pollutants. J. Clean. Prod..

[171] Oladimeji T. (2024). Review on Heavy Metals from Industrial Wastewater. Heliyon.

[172] Correia C. (2022). Fluorescent Nanosensor for Ag^+^ and Hg^2+^ Using Eu-Doped Carbon Dots. Nanomaterials.

[173] Ma Y., Song Y., Ma Y., Wei F., Xu G., Cen Y., Shi M., Xu X., Hu Q. (2018). N-Doped Carbon Dots as a Fluorescent Probe for the Sensitive Detection of Carbamazepine Based on the Inner Filter Effect. New J. Chem..

[174] Li Y.-Y. (2024). Single-Atom Iron Doped Carbon Dots with Highly Efficient Electrochemiluminescence for Ultrasensitive Detection of MicroRNAs. Anal. Chem..

[175] Sarkar T., Bohidar H., Solanki P. (2018). Carbon Dots-Modified Chitosan Electrochemical Biosensor for Vitamin D. Int. J. Biol. Macromol..

[176] Achadu O. (2021). Sulfur-Doped Carbon Dots@polydopamine Magnetic Silver Nanocubes for Norovirus. Biosens. Bioelectron..

[177] Chunduri L.A. (2016). Carbon Dot Based Microplate and Microfluidic Chip Immunoassay for HIV-1 P24 Antigen. Microfluid. Nanofluid..

[178] Alarfaj N.A. (2020). Immunosensing-Fluorescence Detection of CYFRA 21-1 via Carbon Quantum Dots/Zinc Oxide Nanocomposite. Nanoscale Res. Lett..

[179] Wu L. (2013). Ultrasensitive electrochemiluminescence immunosensor for tumor marker detection based on nanoporous sliver@carbon dots as labels. Sens. Actuators B.

[180] Liu H., Zhou X., Shen J., Xing D. (2017). Sensitive Detection of Hg^2+^ with Switchable Electrochemiluminescence Luminophore and Disposable Bipolar Electrode. ChemElectroChem.

[181] Pourmadadi M., Nouralishahi A., Shalbaf M., Shabani Shayeh J., Nouralishahi A. (2023). An Electrochemical Aptasensor for Detection of Prostate-Specific Antigen Based on Carbon Quantum Dots–Gold Nanoparticles. Biotechnol. Appl. Biochem..

[182] Filik H. (2022). Electrochemical immunosensor for individual and simultaneous determination of Cytokeratin fragment antigen 21-1 and Neuron-specific enolase using carbon dots-decorated multiwalled carbon nanotube electrode. Microchem. J..

[183] Chen B., Li L., Yang Q., Zhang M. (2023). Self-Corrected Dual-Optical Immunosensors Using Carbon dots@SiO_2_@MnO_2_ Improving Diethyl Phthalate Detection Accuracy. Talanta.

[184] Morrison D.W.G., Dokmeci M.R., Demirci U., Khademhosseini A. (2008). Clinical Applications of Micro- and Nanoscale Biosensors. Biomed. Nanostruct..

[185] Tricoli A., Neri G. (2018). Miniaturized Bio-and Chemical-Sensors for Point-of-Care Monitoring of Chronic Kidney Diseases. Sensors.

[186] Kulkarni M.B., Ayachit N.H., Aminabhavi T.M. (2023). A Short Review on Miniaturized Biosensors for the Detection of Nucleic Acid Biomarkers. Biosensors.

[187] Liu Y., Zhao X., Liao M., Ke G., Zhang X.-B. (2024). Point-of-Care Biosensors and Devices for Diagnostics of Chronic Kidney Disease. Sens. Diagn..

[188] Wang X., Li F., Guo Y. (2020). Recent Trends in Nanomaterial-Based Biosensors for Point-of-Care Testing. Front. Chem..

[189] Zhang J., Zhang X., Zhang Y. (2025). Emerging Biosensors Integrated with Microfluidic Devices: A Promising Analytical Tool for on-Site Detection of Mycotoxins. npj Sci. Food.

[190] Alhalaili B., Popescu I.N., Rusanescu C.O., Vidu R. (2022). Microfluidic Devices and Microfluidics-Integrated Electrochemical and Optical (Bio)Sensors for Pollution Analysis: A Review. Sustainability.

[191] Noviana E., McCord C.P., Clark K.M., Jang I., Henry C.S. (2020). Electrochemical Paper-Based Devices: Sensing Approaches and Progress toward Practical Applications. Lab A Chip.

[192] Hu S.-W., Qiao S., Xu B.-Y., Peng X., Xu J.-J., Chen H.-Y. (2017). Dual-Functional Carbon Dots Pattern on Paper Chips for Fe^3+^ and Ferritin Analysis in Whole Blood. Anal. Chem..

[193] Wang F., Mei L., Qi J., Zhu L. (2024). Paper-based microfluidic sensors utilizing metal–organic framework materials modified with europium and carbon quantum dots for anthrax spore biomarker detection. ACS Appl. Nano Mater..

[194] Kumar P., Sarkar N., Singh A., Kaushik M. (2022). Nanopaper biosensors at point of care. Bioconjug. Chem..

[195] Li W., Zhang X., Miao C. (2020). Fluorescent Paper-Based Sensor Based on Carbon Dots for Detection of Folic Acid. Anal. Bioanal. Chem..

[196] Rossini E.L., Milani M.I., Lima L.S., Pezza H.R. (2021). Paper Microfluidic Device Using Carbon Dots to Detect Glucose and Lactate in Saliva Samples. Spectrochim. Acta Part A Mol. Biomol. Spectrosc..

[197] Zong H., Zhang Y., Liu X., Xu Z., Ye J., Lu S., Guo X., Yang Z., Zhang X., Chai M. (2023). Recent Trends in Smartphone-Based Optical Imaging Biosensors for Genetic Testing: A Review. View.

[198] Zhang D., Liu Q. (2016). Biosensors and Bioelectronics on a Smartphone for Portable Detection. Biosens. Bioelectron..

[199] Öztürk D., Sanko V., Ömeroğlu İ., Khataee A., Demirbaş E., Durmuş M., Yeşilot S., Kılıç Z., Demirbaş Ü., Şenocak A. (2026). Detection of herbicides in food samples using hybrid carbon dot sensors and test kit fabrication via paper-based analytical devices. Microchem. J..

[200] Su D., Han X., Yan X., Jin R., Li H., Kong D., Gao H., Liu F., Sun P., Lu G. (2020). Smartphone-Assisted Robust Sensing Platform for On-Site Quantitation of 2,4-Dichlorophenoxyacetic Acid Using Red Emissive Carbon Dots. Anal. Chem..

[201] Zhu X.B., Yuan X., Han L., Liu H., Sun B. (2021). Smartphone-Integrated Optosensing Platform Based on Red Carbon Dots. Biosens. Bioelectron..

[202] Higgins D.C., Johner C. (2023). Validation of Artificial Intelligence Containing Products Across Regulated Healthcare Industries. Ther. Innov. Regul. Sci..

[203] Adhao V., Ambhore J., Chaudhari S. (2026). Transforming Pharmaceutical Quality Assurance and Validation through Artificial Intelligence. Artif. Intell. Health.

[204] Kavianpour B., Piadeh F., Gheibi M., Ardakanian A., Behzadian K., Campos L.C. (2024). Applications of Artificial Intelligence for Chemical Analysis of Pharmaceuticals in Water. Chemosphere.

[205] Ganthavee V., Trzcinski A.P. (2024). Artificial Intelligence and Machine Learning for Optimization of Pharmaceutical Wastewater Treatment. Environ. Chem. Lett..

[206] Ashkanani Z., Mohtar R., Al-Enezi S., Smith P.K., Calabrese S., Ma X., Abdullah M. (2024). AI-Assisted Review on Remediation of Contaminated Soils with PAHs and Heavy Metals. J. Hazard. Mater..

[207] Xiao M., Xu N., He A., Yu Z., Chen B., Jin B., Jiang L., Yi C. (2023). A Smartphone-Based Fluorospectrophotometer and Ratiometric Fluorescence Nanoprobe for On-Site Quantitation of Pesticide Residue. iScience.

[208] Mintz K.J., Zhou Y., Leblanc R.M. (2019). Recent development of carbon quantum dots regarding their optical properties, photoluminescence mechanism, and core structure. Nanoscale.

[209] de Silva A.P., Gunaratne H.Q.N., Gunnlaugsson T., Huxley A.J.M., McCoy C.P., Rademacher J.T., Rice T.E. (1997). Signaling Recognition Events with Fluorescent Sensors and Switches. Chem. Rev..

[210] Clegg R.M. (2009). Förster Resonance Energy Transfer—FRET What Is It, Why Do It, and How It’s Done. Laboratory Techniques in Biochemistry and Molecular Biology.

[211] Chen S., Jia Q., Zheng X., Mu Q., Zhang R., Chen Y., Liu C., Zhang H. (2018). PEGylated Carbon Dot/MnO_2_ Nanohybrid: A New pH/H_2_O_2_-Driven, Turn-On Cancer Nanotheranostics. Sci. China Mater..

[212] Han C., Park S., Lee D., Jo H., Seo S., Han H., Jeong S., Kwon W. (2026). DNA-Functionalized Nanomaterials for Optical Biosensors. Sens. Actuators Rep..

[213] Luong J.H.T., Vashist S.K. (2020). Chemistry of Biotin–Streptavidin and the Growing Concern of an Emerging Biotin Interference in Clinical Immunoassays. ACS Omega.

[214] Ma Y., Liu D., Xiao W., Ye Y., Zhang L., Chen X., Lv Y., Wu R., Wang L., Li L.S. (2024). A low-cost biotin-streptavidin amplified quantum dot fluorescence immunosensor for enhancing cancer detection and imaging. Microchem. J..

[215] Wang F.T., Wang L.N., Xu J., Huang K.J., Wu X. (2021). Synthesis and modification of carbon dots for advanced biosensing application. Analyst.

[216] Ji C., Zhou Y., Leblanc R.M., Peng Z. (2020). Recent Developments of Carbon Dots in Biosensing: A Review. ACS Sens..

[217] Duran N., Simões M.B., de Moraes A.C.M., Fávaro W.J., Seabra A.B. (2016). Nanobiotechnology of Carbon Dots: A Review. J. Biomed. Nanotechnol..

[218] Das P., Nath P.C., Pandey V.K., Singh R., Rustagi S., Shaikh A.M., Kovács B. (2025). Carbon quantum dots as emerging biosensors for food safety and environmental applications: Advances and challenges. Appl. Food Res..

